# A novel microRNA-182/Interleukin-8 regulatory axis controls osteolytic bone metastasis of lung cancer

**DOI:** 10.1038/s41419-023-05819-8

**Published:** 2023-05-01

**Authors:** Ming-Na Zhao, Ling-Fei Zhang, Zhen Sun, Li-Hua Qiao, Tao Yang, Yi-Zhe Ren, Xian-Zhou Zhang, Lei Wu, Wen-Li Qian, Qiao-Mei Guo, Wan-Xing Xu, Xue-Qing Wang, Fei Wu, Lin Wang, Yutong Gu, Mo-Fang Liu, Jia-Tao Lou

**Affiliations:** 1 Department of Laboratory Medicine, Shanghai Chest Hospital, Shanghai Jiao Tong University, 200030 Shanghai, China; 2 Department of Laboratory Medicine, Shanghai General Hospital, Shanghai Jiao Tong University School of Medicine, 200080 Shanghai, China; 3 Key Laboratory of Systems Health Science of Zhejiang Province, School of Life Science, Hangzhou Institute for Advanced Study, University of Chinese Academy of Sciences, 310024 Hangzhou, China; 4 State Key Laboratory of Molecular Biology, State Key Laboratory of Cell Biology, Shanghai Key Laboratory of Molecular Andrology, Shanghai Institute of Biochemistry and Cell Biology, Center for Excellence in Molecular Cell Science, Chinese Academy of Sciences, University of Chinese Academy of Sciences, 200030 Shanghai, China; 5 School of Life Science and Technology, Shanghai Tech University, 201210 Shanghai, China; 6 Department of Orthopaedic Surgery, Zhongshan Hospital, Fudan University, 200030 Shanghai, China

**Keywords:** Lung cancer, Metastasis

## Abstract

Bone metastasis is one of the main complications of lung cancer and most important factors that lead to poor life quality and low survival rate in lung cancer patients. However, the regulatory mechanisms underlying lung cancer bone metastasis are still poor understood. Here, we report that microRNA-182 (miR-182) plays a critical role in regulating osteoclastic metastasis of lung cancer cells. We found that miR-182 was significantly upregulated in both bone-metastatic human non–small cell lung cancer (NSCLC) cell line and tumor specimens. We further demonstrated that miR-182 markedly enhanced the ability of NSCLC cells for osteolytic bone metastasis in nude mice. Mechanistically, miR-182 promotes NSCLC cells to secrete Interleukin-8 (IL-8) and in turn facilitates osteoclastogenesis via activating STAT3 signaling in osteoclast progenitor cells. Importantly, systemically delivered IL-8 neutralizing antibody inhibits NSCLC bone metastasis in nude mice. Collectively, our findings identify the miR-182/IL-8/STAT3 axis as a key regulatory pathway in controlling lung cancer cell-induced osteolytic bone metastasis and suggest a promising therapeutic strategy that targets this regulatory axis to interrupt lung cancer bone metastasis.

## Introduction

Lung cancer is the leading cause of cancer-related death worldwide, causing ∼18% of cancer-related deaths [1]. More than 65% of lung cancer patients have local or distant metastasis at diagnosis, with bone metastasis as the most prevalent malignant clinical symptom [2]. In particular, approximately 30–40% of non-small cell lung cancer (NSCLC) patients develop bone metastasis, with an average survival rate of about 6 months [3, 4]. However, effective intervention strategies for lung cancer bone metastasis are still lacking due to a poor understanding of the regulatory mechanisms.

Recent studies have advanced our understanding of the process of breast and prostate cancer bone metastasis. Bone-metastatic breast and prostate cancer cells are shown to secrete various inflammatory factors or growth factors, including IL-11, IL-6, IL-8, TGF-β, TNF, and EGF, and directly activate osteoclast progenitor cells [5–8]. Moreover, recent studies on breast cancer cells report that tumor-derived Jagged1 promotes osteoclast formation and bone absorption by activating the Notch signaling pathway in osteoclasts [9], while cancer cell secreted-IL-11 functions as a pro-osteolytic factor by activating the JAK1/STAT3 pathway in osteoclast progenitor cells [10, 11]. In addition, prostate cancer cell-secreted PTHrP may stimulate the differentiation and maturation of osteoclast cells by altering the homeostasis between the osteoclastogenesis inducer RANKL and its decoy receptor Osteoprotegerin (OPG) in metastatic niches [12–15]. These processes together promote osteoclast differentiation in metastatic niches, thereby increasing osteolytic bone lesions and facilitating the bone metastasis of breast and prostate cancer cells. Similarly, more than 70% of lung cancer bone metastasis display osteolytic bone destruction [16]. However, unlike recent advances in our understanding of breast and prostate cancer bone metastasis, how lung cancer bone metastasis is regulated remains largely unexplored.

MicroRNAs (miRNAs) are known as an emerging class of post-transcriptional gene regulators in eukaryotic cells, which have been shown to play critical roles in all stages of tumor progression [17, 18]. In particular, miRNAs play a critical role in modulating cellular pathways implicated in osteolytic bone destruction and expression of key cytokines and/or chemokines in bone microenvironment [19–21]. For instance, overexpression of miR-155 in RAW264.7 cells blocks osteoclastogenesis by repressing MITF and PU.1, two crucial transcription factors for osteoclast differentiation [22], while breast cancer cell-induced miR-141 and miR-219 downregulation in osteoclasts promote osteoclast differentiation and osteolytic bone metastasis [23]. Our recent study also showed that NSCLC cell-derived exosomal miR-17-5p promote osteoclast differentiation by targeting *PTEN* [24], but it has been largely unknown whether and how miRNAs in lung cancer cells are directly involved in regulating the osteolytic bone metastasis.

In the present study, we discovered that miR-182 acts as a critical regulator in controlling lung cancer bone metastasis. This miRNA was previously reported to regulate tumor occurrence, progression, and distant metastasis in various types of cancers, including melanoma [25], breast cancer [26], and lung cancer [27, 28]. We found that miR-182 was upregulated in bone-metastatic NSCLC cells and tumors and further showed that this miRNA significantly promoted the osteolytic bone metastasis of NSCLC cells in nude mice. Our mechanistic studies revealed that miR-182 enhanced IL-8 expression in NSCLC cells by targeting the NF-κB signaling inhibitor gene *KLHL21* [29], and thus increased IL-8 secretion from NSCLC cells to facilitate osteoclastogenesis via activating STAT3 signaling in osteoclast progenitor cells. Collectively, our findings indicate the miR-182/IL-8/STAT3 cascade as a novel regulatory axis in controlling osteoclast differentiation and osteolytic lesion development in metastatic niches, providing new mechanistic insights into lung cancer bone metastasis and potential therapeutic targets for the treatment of lung cancer bone metastasis.

## Materials and methods

### RNA oligonucleotides and plasmids

miR-182 mimics, anti-miR-182, and their cognate negative control RNAs were purchased from RiboBio (Guangzhou, China). Human *KLHL21* coding sequences were cloned into the p3×Flag-CMV-14 expression vector (Sigma) to construct p3×Flag-KLHL21. For reporter pRL-TK-KLHL21 3′-untranslated region (3′-UTR), the human KLHL21 3′-UTR was cloned downstream of the Renilla luciferase gene in pRL-TK (Promega). Eight nucleotides in KLHL21 3′-UTR corresponding to 5′ part of miR-182 were deleted in the pRL-KLHL21 3′-UTR Mut construct. All constructs were confirmed by DNA sequencing.

### Cell lines, cell infection, and transfection

Human NSCLC cell line A549 and murine pre-osteoclast cell line RAW264.7 were obtained from the American Type Culture Collection (ATCC) and cultured according to their guidelines. All cell lines have been authenticated using STR profiling. Mycoplasma contamination testing was performed and the cells were proved to be mycoplasma free. A549 subline stably expressing luciferase (referred to as A549-luc) was generated from the parental cell line, as previously reported [30, 31]. Generation of the bone-metastatic A549 subline was carried out as previously reported [10, 32]. In brief, A549-luc cells were inoculated into the left cardiac ventricle of BALB/c athymic nude mice (6–8 weeks old), and then the bone tissue-resided A549-luc cells were isolated from mouse bones indicated by Bioluminescence imaging (BLI). After in vitro cultivation, the isolated A549-luc cells were administered again into the left cardiac ventricle. After three cycles of selection, the obtained A549-luc subline was defined as bone-metastatic subline (referred to as A549-BM; Fig. S1A, B).

The pseudovirus of GV369-miR-182 and control viruses were purchased from GENECHEM (Shanghai, China). NSCLC cells were infected with indicated viruses and subjected to antibiotic selection for enrichment before the assays. Cell transfection was performed using Lipofectamine 2000 (Invitrogen) according to the manufacturer’s instruction. For RNA oligonucleotide transfection, 50 nmol/L of miRNA mimics and 100 nmol/L of antisense oligonucleotides were used.

### RNA isolation and real-time quantitative PCR (qPCR)

The assays were performed as we described previously [17, 18]. Total RNAs were extracted from cells or tissues with TRIzol reagent (Invitrogen). miRNA and mRNA levels were quantified by quantitative reverse transcription PCR (qPCR) using SYBR Green (Takara), with U6 small nuclear RNA and β-actin as internal normalized references, respectively. The qPCR results were analyzed and shown as relative miRNA or mRNA levels of the CT (cycle threshold) values, which were then converted as fold change. The primer sequences for qPCR were provided in Table S1.

### Small RNA sequencing and transcriptome sequencing analyses

For small RNA sequencing, total RNAs were isolated from cells or tissues using TRIzol reagent (Invitrogen) according to the manufacturer’s protocol. The quantity and integrity of RNAs were assessed by using the K5500 and the Agilent 2200 TapeStation (Agilent Technologies, USA), respectively. Briefly, RNAs were ligated with 3′ RNA adapter, and followed by 5′ adapter ligation. Subsequently, the adapter-ligated RNAs were subjected to RT-PCR and amplified with a low cycle. Then the PCR products were size selected by PAGE gel according to instructions of NEBNext® Multiplex Small RNA Library Prep Set for Illumina® (Illumina, USA). The purified library products were evaluated using the Agilent 2200 TapeStation. The libraries were sequenced by HiSeq 2500 (Illumina, USA) with single-end 50 bp at Ribobio Co. Ltd (Ribobio, China).

RNA sequencing and initial data analyses were conducted by LC Sciences using illumina Novaseq™ 6000 and 2 × 150 bp paired-end sequencing. Gene Ontology (GO) annotations are based on UniProtBlast, Entrez Gene, and Expasy Proteomic databases. Differential gene expression was assessed through average reads of multiple RNA samples, with the cutoff for differentially expressed genes set as fold change >1.5.

### Immunoblotting and immunofluorescent assays

The assays were carried out as we recently described [17]. For detecting protein levels in conditioned medium, the supernatants were collected, quantified and denatured for western blot analysis as described previously [33, 34]. The primary antibodies used in this study were the antibodies for anti-p-STAT3 (9145 T, Cell Signaling Technology), STAT3 (#4904, Cell Signaling Technology), NFATc1 (sc-7294, Santa Cruz Biotechnology), p-P65 (#3033, Cell Signaling Technology), P65 (#8242, Cell Signaling Technology), p-ERK (#4370, Cell Signaling Technology), ERK (#4695, Cell Signaling Technology), p-PI3K (#4228, Cell Signaling Technology), PI3K (#4249, Cell Signaling Technology), β-actin (A3854; Sigma-Aldrich), IL-8 (MAB208-100, R&D Systems), IL-1 (AF-200-SP, R&D Systems), IL-12 (AF-219-SP, R&D Systems), CXCL3 (MAB276-SP, R&D Systems), KLHL21(GTX120580, GeneTex). Western blot images were captured using a Tanon 5200 chemiluminescent imaging system. Multiplex immunofluorescence staining was prepared as described previously [35]. Bone samples were respectively stained with primary antibodies, including the antibodies for p-STAT3 (9145T, Cell Signaling Technology), CTSK (ab300569, Abcam), MMP9 (10375-2-AP, Proteintech), and CK8 (ab53280, Abcam). Biotinylated secondary antibody was used with ABC Kit (ZSGB-BIO) and TSA detection kit (Invitrogen) to indicate the positively stained cells, with nuclei counterstained with DAPI (Sigma).

### Histological analysis of bone tissues

The experiment was carried out as previously described [23]. In brief, the dissected femurs were subjected to fixation in 4% paraformaldehyde for 48 h followed by decalcification through 2 weeks of incubation with daily changed 15% tetrasodium EDTA. Thereafter, bone tissues were dehydrated with a graded series of ethanol, immersed into xylene, paraffin-embedded, and then cryo-sectioned in the coronal plane at a thickness of 8 μm.

### ELISA analysis

Quantitative levels of cytokines in the conditioned medium of cultured cells were determined by ELISA according to the manufacturer’s protocol. The ELISA kit are as follows: IL-8 (EK0413, Boster Biological Technology), IL-12 (EK0421, Boster Biological Technology), IL-1 (EK0389, Boster Biological Technology), CXCL3 (EK1364, Boster Biological Technology).

### Bioluminescence imaging (BLI)

Bioluminescence imaging was performed as described previously [9, 11]. In brief, tumor-bearing mice were anesthetized and then administered intraperitoneally with D-Luciferin (75 mg/kg of body weight). After 10 min, a Perkin Elmer IVIS Imaging System was employed to acquire bioluminescence images. The imaging acquisition time was set to 60 seconds initially and decreased along with signal strength during the time course for avoiding saturation. Measurement of the photon flux within the interested region delineated around the BLI signals was conducted by using the Living Image software.

### In vitro osteoclast differentiation and Tartrate-resistant acid phosphatase (TRAP) staining

For RANKL treatment, RAW264.7 cells were treated with indicated concentration of RANKL (Peprotech, 20 ng/ml) or Osteoprotegerin (OPG; Peprotech, 20 ng/ml) with media changed every 2 days. For tumor-conditioned media (CM), CM was collected from the sub-confluent cancer cells with indicated treatments. The CM was passed through a 0.22 μm filter before addition to RAW264.7 cells. For antibody blocking assay, antibodies against IL-8 (R&D Systems, MAB208-100, 5 µg/mL), IL-1 (R&D Systems, AF-200-SP, 5 µg/mL), IL-12 (R&D Systems, AF-219-SP, 5 µg/mL), or CXCL3 (R&D Systems, MAB276-SP, 0.5 µg/mL) were respectively added to the CM from miR-182-expressing A549 cells. For stimulation assay, recombinant human IL-8, IL-1, IL-12, and CXCL3 proteins were respectively added to the CM from A549 cells. RAW264.7 cells were cultured with the indicated CM, using fresh CM replaced daily for 6 days. At the treatment end, TRAP staining (Wako, Osaka, Japan) was performed to examine the osteoclast differentiation. For cultured cells, TRAP^+^ multi-nucleated cells were scored as mature osteoclasts. For the bone metastatic tissue section, the number of osteoclasts in metastatic niches was assessed as TRAP^+^ cells along the tumor-bone interface and presented as the number/mm of interface.

### Bone metastasis assay and therapeutic experiments

Both intracardiac (IC) injection and intrailiac artery (IIA) injection were used to generate the mouse model for NSCLC bone metastasis. For IC injection, 5 × 10^5^ NSCLC cells suspended in PBS were injected into the left ventricle of anesthetized BALB/c athymic nude mice (6–8 weeks old) as described previously [9, 36]. For IIA injection, 1 × 10^5^ NSCLC cells suspended in PBS were injected into external iliac artery using 31 G needles as described previously [37, 38]. For IL-8 neutralizing antibodies treatment, 3 days after IIA injection of miR-182-overexpressing A549 cells, IL-8 neutralizing antibodies (R&D Systems, MAB208-100) or IgG (R&D Systems, 1-100-A) in PBS (0.1 mg/mouse) were administrated intravenously twice a week for three consecutive weeks (Fig. 6A). Tumor burden was measured by Bioluminescence imaging (BLI) after the completion of treatment. Animals were then sacrificed and the hindlimbs were resected and subjected to histological and morphometric analyses. Hindlimbs bearing metastatic tumors were scanned using a Micro-CT scanner (Model 1272; Skyscan) for detecting bone damage. Five mice were included for each experimental group, and each experiment was repeated 3 times.

### Statistical analyses

Data were shown as mean ± SEM of three independent experiments performed in triplicate. Comparison between paired treated groups and controls was made using Student’s *t* test. *P*-values were compared between the two groups with a value <0.05 (denoted by asterisks) considered significant. Pearson’s correlation analysis was used to calculate the correlation between the two groups. Kaplan–Meier survival analysis was used to depict the survival of patients between different groups.

### Study approval

Primary NSCLC tumor specimens, non-tumorous adjacent tissues and metastatic bone specimens were collected during surgery from Shanghai Chest Hospital Affiliated to Shanghai Jiao Tong University and Zhongshan Hospital Affiliated to Fudan University (Shanghai, China) with written informed consent from patients. The specimens were immediately subjected to snap freezing followed by storage at −80 °C. The sample collection was approved by the Medical Ethical Committee of the hospitals. All animal experiments were performed under the protocols approved by Shanghai Institute of Biochemistry and Cell Biology, Chinese Academy of Sciences and in accordance with the Guide for the Care and Use of Laboratory Animals (NIH publication nos. 80-23, revised 1996).

## Results

### miR-182 is upregulated in bone-metastatic NSCLC cells and tumors

**Fig. 1:**
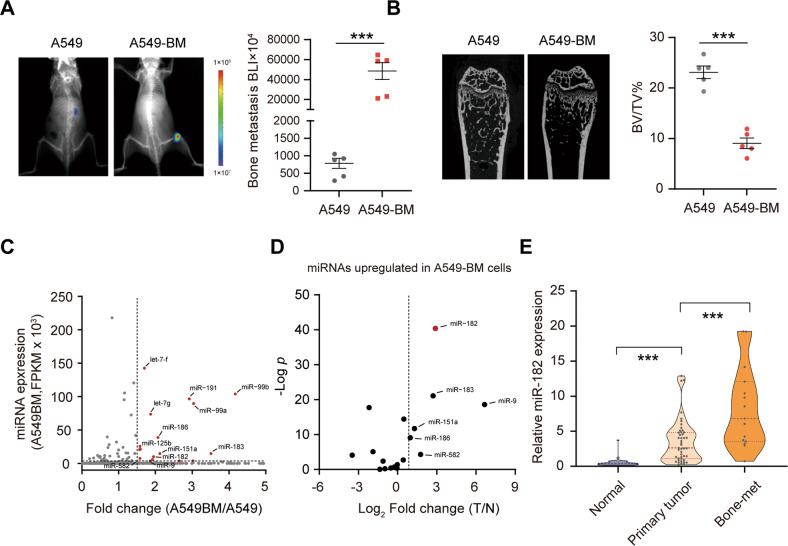
miR-182 is upregulated in bone-metastatic NSCLC cell line and tumor specimens. **A**, **B** Bioluminescence imaging (BLI, **A**) and micro-CT (**B**) analyses of bone metastasis in A549 or A549-BM cell-injected mice. On 30 days post injection of A549 or A549-BM cells, the bioluminescence signals in bone-metastatic tumors were detected by BLI (**A**), and bone damage was measured by micro-CT and quantified by bone volume/tissue volume (BV/TV) (B). **C**, **D** Identification of candidate miRNAs critical for lung cancer bone metastasis. **C** Scatter plot showing the relative expression of miRNAs in A549-BM cells compared with parental A549 cells, with the 17 upregulated and also abundantly expressed miRNAs in A549-BM cells indicated in red. **D** Scatter plot showing the relative expression of the 17 miRNAs in NSCLC specimens compared with adjacent normal lung tissues using StarBase and ATCG datasets, with *P*-value (FDR) in the *Y*-axis and highlighted significantly elevated miRNAs. The vertical dashed lines indicated fold-change cutoff at 1.5. (**E**) Quantification of the miR-182 level in primary NSCLC tumors (Primary tumor, *n* = 45), non-tumor lung tissues (Normal, *n* = 45), and bone metastatic tumors from NSCLC patients (Bone-met, *n* = 15) using qPCR. The average values±SEM of three separate experiments are plotted. ***P < 0.001.

To facilitate our studies of lung cancer bone metastasis, we first established a murine model of bone metastasis as described previously [10] and generated a bone-metastatic subline of human NSCLC cell line A549 (referred to as A549-BM; Fig. S1A, B). By bioluminescence imaging (BLI), we verified that A549-BM subline showed a much higher ability for bone metastasis in nude mice relative to parental A549 cell line (Fig. 1A). Micro-CT scan further showed much more serious bone damage in A549-BM cell-implanted mice compared with controls (Fig. 1B). These results together indicate that A549-BM subline is prone to bone metastasis and causes osteolytic lesions similar to clinical symptoms of lung cancer bone metastasis patients.

To gain new insights into the regulatory mechanisms underlying lung cancer bone metastasis, we performed small RNA sequencing and found that a total of 17 abundantly expressed miRNAs (read counts > 2000) were upregulated (fold-change cutoff at 1.5) in A549-BM cells relative to the parental controls (Fig. 1C). We next compared their expression in NSCLC tumor specimens relative to adjacent normal lung tissue controls from both StarBase and Cancer Genome Atlas (TCGA) databases, and found that only 6 out of the 17 upregulated miRNAs in A549-BM cells were elevated (fold change >1.5) in tumor tissues (Fig. 1D). Among these, miR-182 stuck our attention, given that it is most significantly elevated in NSCLC tumors relative to normal controls (Fig. 1D). By qPCR, we further compared miR-182 expression in a cohort of human NSCLC non-tumorous adjacent tissues, primary tumors, and bone metastatic tumors, and found that miR-182 expression was significantly elevated in primary NSCLC tumors and further elevated in metastatic bone tumors (Fig. 1E). These results together imply that miR-182 might be involved in regulating lung cancer bone metastasis.

Additionally, we found that the miR-182 level in primary tumors from the patients with metastasis was significantly higher than that from the patients without metastasis (Fig. S2A). By Kaplan–Meier analysis, we found that patients with higher miR-182 expression in tumors have significantly shorter overall survival (Fig. S2B). Consistent with our findings, miR-182 expression is also significantly elevated in melanoma and bladder tumors from metastatic patients [25, 39]. These findings together suggest that miR-182 might be considered as a potentially metastatic biomarker of pan-cancers.

### miR-182 promotes lung cancer bone metastasis in mice

**Fig. 2:**
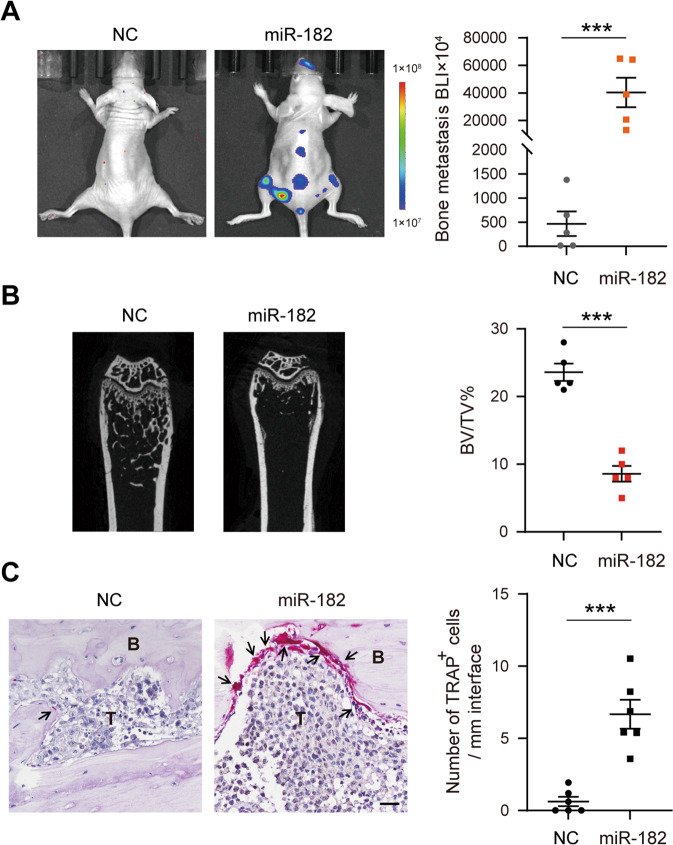
miR-182 promotes lung cancer bone metastasis in mice. **A**, **B** miR-182 overexpression potently increased the bone-metastatic ability of A549 cells in nude mice. On 4 weeks post-IC injection of negative control (NC) or miR-182-expressing lentivirus (miR-182)-infected A549 cells, the bioluminescence signals of bone-metastatic tumors were detected by BLI (**A**), and bone damage was measured by micro-CT and quantified by BV/TV (**B**). **C** TRAP staining analysis of osteoclast formation at the boundary between bone and metastatic tumor in NC or miR-182 expression A549 cell-injected mice, with TRAP-positive osteoclasts indicated by arrowheads. Scale bars, 50 μm. The average values ± SEM of three separate experiments are plotted. ***P < 0.001.

To experimentally determine whether miR-182 is functionally important for NSCLC cells to metastasize to bones, we elevated miR-182 expression in parental A549 cells through infection with miR-182-expressing lentivirus (Fig. S3A) and subsequently injected the cells through the intracardiac route of BALB/c athymic nude mice (Fig. S3B). 4 weeks post injection, our BLI identified a potent increase of metastatic burden in both hindlimb bones and spines in miR-182-overexpressing A549 cell-injected mice compared with the negative control (NC) group (Fig. 2A). By micro-CT assay, we found more severe osteolysis in miR-182-overexpressing A549 cell-injected mice compared with the NC group (Fig. 2B). Moreover, Tartrate-resistant acid phosphatase (TRAP) staining and immunohistochemical staining showed a marked increase of osteoclasts at the tumor-bone boundary in the miR-182-overexpressing metastatic tumors (Fig. 2C). By co-staining of MMP9 and CTSK, and K8, we found that MMP9 level was elevated in CTSK-positive cells at the tumor-bone boundary of the miR-182-OE lesions compared with the control group (Fig. S3C), further confirming the increase of osteoclastogenesis at the tumor-bone boundary. These results together indicate that miR-182 overexpression promotes the osteolytic bone metastasis of NSCLC cells in nude mice, suggesting a functional role of miR-182 in regulating lung cancer bone metastasis.

### miR-182 promotes the NSCLC cell-induced osteoclast differentiation

**Fig. 3:**
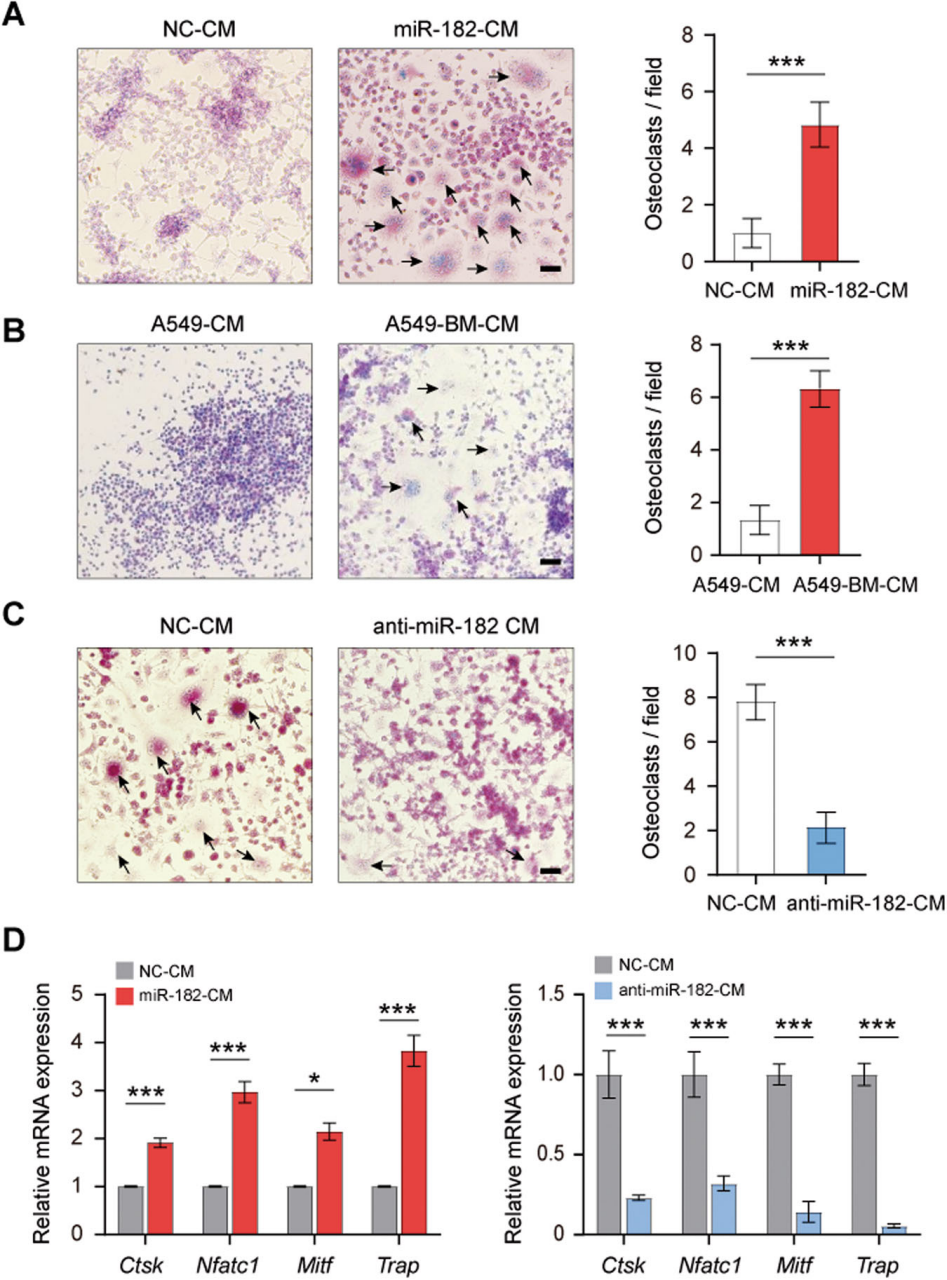
Conditioned media (CM) from miR-182-upregulated NSCLC cells promotes osteoclast precursor cell differentiation. **A**–**C** TRAP staining analysis of the osteoclast differentiation of RAW264.7 cells after cultured in the CM from miR-182 or NC overexpressing A549 cells (**A**), A549-BM or parental A549 cells (**B**), NC or anti-miR-182-transfected A549-BM cells (**C**) for 6 days. Arrowheads indicate mature, multi-nucleated osteoclasts. **D** qPCR-based analysis of the effect of indicated CM on expression of osteoclastogenesis-related genes in RAW264.7 cells. Scale bars, 50 μm. The average values ± SEM of three separate experiments are plotted. *P < 0.05; ***P < 0.001.

Our above results showed that administration of miR-182-overexpressing NSCLC cells substantially enhanced osteolysis in metastatic niches, leading us to hypothesize that miR-182 might facilitate cancer cell-induced osteoclastogenesis. To test this hypothesis, we cultured murine pre-osteoclast RAW264.7 cells in conditioned media (CM) collected from NSCLC cells A549 or H1299 infected with miR-182-expressing (miR-182-CM) or NC lentivirus (NC-CM), respectively. As a positive control, RANKL, a potent inducer of osteoclastogenesis [23], effectively drove RAW264.7 cell differentiation into mature, multi-nucleated osteoclasts (Fig. S4A). Intriguingly, our ELISA assay showed a similar level of RANKL in the CM from control A549, miR-182-overexpressing A549, and A549-BM cells (Fig. S4B), but we found that miR-182-CM, but not NC-CM, markedly induced the differentiation of RAW264.7 cells into mature osteoclasts (Figs. 3A andS4C). Consistently, we found that the CM from A549-BM cells, which have a higher endogenous miR-182 expression, was much more effective in driving RAW264.7 cell differentiation compared with that from parental A549 cells (Fig. 3B). These results support a role of miR-182 in NSCLC cell-induced osteoclastogenesis. To corroborate this, we inhibited miR-182 function in A549-BM cells by anti-miR-182 transfection. As expected, inhibition of miR-182 function significantly overrode the stimulatory effect of A549-BM CM on osteoclast differentiation of RAW264.7 cells (Fig. 3C). Indeed, several key regulator genes for osteoclast differentiation, including *Ctsk*, *Nfatc1*, *Mitf*, and *Trap* were significantly upregulated in the miR-182-CM-treated RAW264.7 cells and downregulated in the anti-miR-182-CM-treated cells compared with control treatment (Figs. 3D and S4D). Moreover, we found that miR-182-CM treatment barely altered the proliferation, apoptosis, and migration of RAW264.7 cells compared with control treatment (Fig. S4E–G). These results together suggest that miR-182 is able to promote the NSCLC cell-induced osteoclast differentiation.

### miR-182 in NSCLC cells enhances IL-8 secretion and in turn promotes osteoclast differentiation

**Fig. 4:**
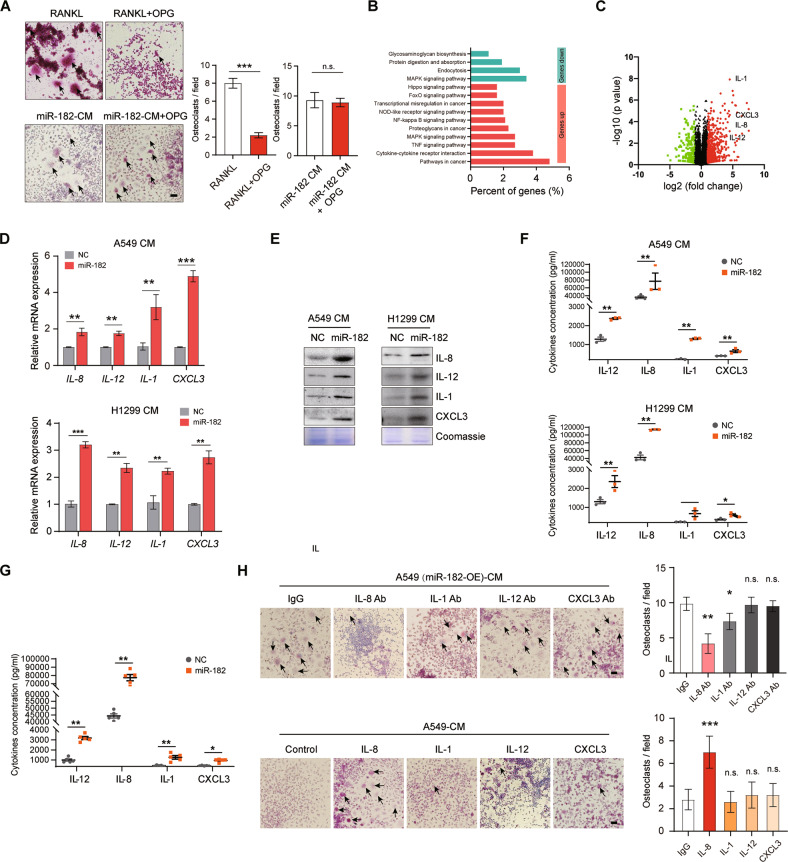
miR-182 upregulated NSCLC cells promote osteoclastogenesis through enhancing IL-8 secretion. **A** TRAP staining analysis of the osteoclast differentiation of RAW264.7 cells after cultured in the indicated CM for 6 days. Arrowheads indicate mature, multi-nucleated osteoclasts. **B** Transcriptome-seq combined with Gene Ontology analysis revealed the enrichment of multiple signaling pathways in miR-182-overexpressing A549 cells. **C** Volcano plot showing differentially expressed genes in miR-182-overexpressing A549 cells compared with the negative control cells, with selected cytokines highlighted. **D** qPCR analysis of the mRNA expression of *IL-8, IL-1, IL-12 and CXCL3* in miR-182-overexpressing A549 (left) or H1299 cells relative to controls (right). **E**, **F** Western blotting of IL-8, IL-1, IL-12, and CXCL3 levels in CM from miR-182-overexpressing (left) or H1299 cells (right) relative to controls, with total protein staining (Coomassie) confirmed equal loading. **F**, **G** ELISA analysis of IL-8, IL-1, IL-12, and CXCL3 levels in the CM from miR-182-overexpressing (top) or H1299 cells (bottom) relative to controls (**F**) or in blood samples collected from mice bearing bone metastases developed by miR-182-overexpressing A549 cells or NC A549 cells (**G**). (H) TRAP staining analysis of the effects of depletion of indicated factors in the miR-182-CM (top) or their addition to the CM from A549 cells (bottom) on osteoclast differentiation of RAW264.7 cells. Arrowheads indicate mature, multi-nucleated osteoclasts. Scale bars, 50 μm. The average values ± SEM of three separate experiments are plotted. n.s., not significant; *P < 0.05; **P < 0.01; ***P < 0.001.

We next asked how miR-182 in NSCLC cells stimulates the differentiation of pre-osteoclast cells. Consistent with our observation of a similar level of RANKL in the CM from control A549 and miR-182-overexpressing A549 (Fig. S4B), TRAP staining showed that Osteoprotegerin (OPG), a decoy receptor for RANKL [40], barely altered the stimulatory effect of miR-182-CM on osteoclast differentiation (Fig. 4A). In sharp contrast, OPG effectively attenuated the RANKL-induced RAW264.7 cell differentiation (Fig. 4A). These results suggest that miR-182 in NSCLC cells drives osteoclastogenesis in a RANKL-independent manner.

To further explore the molecular mechanisms underlying miR-182 action on lung cancer bone metastasis, we next performed transcriptome-seq analysis of the miR-182-expressing or NC lentivirus-infected A549 cells. Pathway enrichment analysis of differentially expressed genes revealed significant enrichment of cytokine and cancer-related pathways in the miR-182-overexpressing A549 cells (Fig. 4B). In particular, several well-known inflammatory factors in tumor microenvironment, including IL-8, IL-1, IL-12, and CXCL3, were significantly elevated in the miR-182-overexpressing cells relative to NC-infected cells (Fig. 4C). Our qPCR confirmed that IL-8, IL-1, IL-12, and CXCL3 were significantly upregulated in either miR-182-overexpressing A549 and H1299 cells (Fig. 4D) or endogenous miR-182 highly expressing A549-BM cells (Fig. S5A). Moreover, both western blotting and ELISA assays verified higher protein levels of these cytokines in miR-182-CM relative to NC-CM (Fig. 4E, F). These results together support that miR-182 facilitates the expression of these cytokines in NSCLC cells. In line with this, we found higher serum levels of these cytokines in miR-182-overexpressing A549 cell-injected mice compared with the control group (Fig. 4G). As expected, miR-182 level was significantly elevated in the EVs isolated from A549-BM cells, miR-182-overexpressing A549 cells or the blood of mice injected with miR-182-overexpressing A549 cells compared with the respective controls (Fig. S5B–D). This suggests that miR-182 could be used as a circulating biomarker for the early detection of bone metastasis. Collectively, these results suggest IL-8, IL-1, IL-12, and CXCL3 as candidate effectors for miR-182-CM action on osteoclastogenesis.

To examine whether these candidate effectors are functionally required for miR-182-CM-induced osteoclastogenesis, we respectively depleted them in the miR-182-CM via antibody blocking. Interestingly, the neutralizing antibody for IL-8, but not those for IL-1, IL-12, and CXCL3, effectively attenuated the stimulatory effect of miR-182-CM on RAW264.7 cell differentiation (Fig. 4H). Conversely, supplementation of recombinant IL-8, rather than IL-1, IL-12, and CXCL3, in the CM from parental A549 cells, remarkedly promoted the differentiation of RAW264.7 cells (Fig. 4H). These results suggest that IL-8 is an authentic effector responsible for miR-182-CM-induced osteoclastogenesis. Consistently, our ELISA assay confirmed that IL-8 level was potently elevated in the CM form miR-182-expressing lentivirus-infected A549 cells and reduced in the CM form anti-miR-182 transfeced-A549-BM cells (Fig. S5E). These results together suggest that miR-182 promotes osteoclast differentiation likely via enhancing NSCLC cells to secrete IL-8 in the tumor microenvironment.

### miR-182 in NSCLC enhances IL-8 expression and secretion via targeting KLHL21

**Fig. 5:**
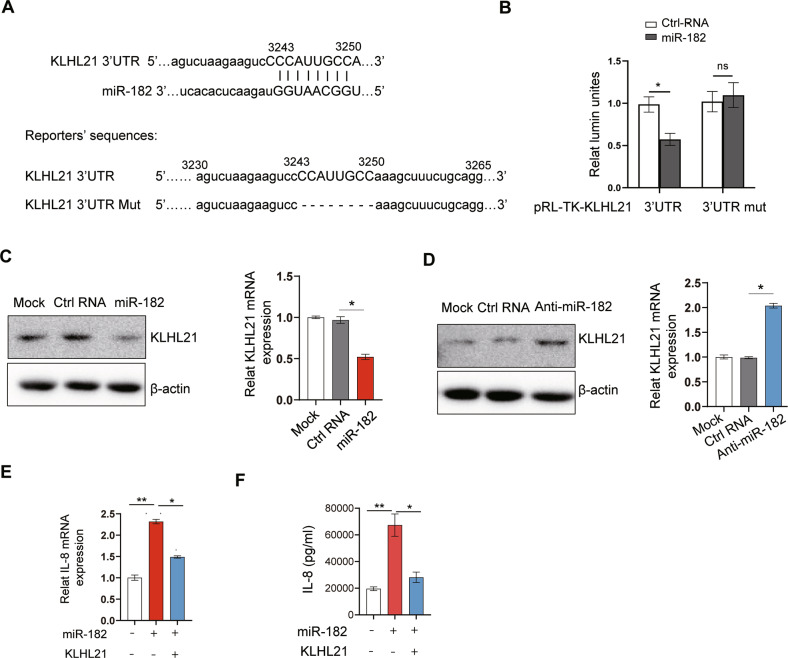
miR-182 enhances *IL-8* expression in NSCLC cells through targeting KLHL21. **A** *KLHL21* was predicted to be a target of miR-182. Top, predicted miR-182 regulatory elements (seed sequences in upper case) in *KLHL21* 3′UTR. Bottom, sequences of the wild-type (pRL-TK-KLHL21 3′UTR) and the mutated (pRL-TK-KLHL21 3′UTR mut) *KLHL21* 3′UTR luciferase reporters. **B** Luciferase reporter assay for the *KLHL21* 3′UTR reporter in 293T cells. **C** Western blot and qPCR analyses of KLHL21 protein (left) or mRNA levels (right) in miR-182-overexpressing A549 cells. **D** Western blot and qPCR analyses of KLHL21 protein (left) or mRNA levels (right) in anti-miR-182–transfected A549 cells. **E**, **F** qPCR (**E**) and ELISA (**F**) analyses of the effect of ectopic expression of KLHL21 on *IL-8* expression and secretion in miR-182-overexpressing A549 cells. The average values ± SEM of three separate experiments are plotted. *P < 0.05; **P < 0.01; n.s., not significant.

We next asked how miR-182 enhances NSCLC cells to secrete IL-8. To this end, we first used computational prediction programs [41, 42] to predict miR-182 targets. Interestingly, we found that *KLHL21*, which encodes an inhibitor of NF-κB signaling pathway [29], was predicted to be a target of miR-182 (Fig. 5A, top). Given that NF-κB signaling has been well-known for its role in regulating *IL-8* expression [43, 44], we hypothesized that miR-182 might elevate *IL-8* expression in NSCLC cells via regulating the KLHL21:NF-κB axis. To begin to test this hypothesis, we first experimentally tested whether miR-182 regulates *KLHL21*. We constructed luciferase reporters by cloning the wild-type 3′-untranslated regions (UTR) of *KLHL21* or its mutant version (with deletion of the 8-bp sequence complementary to the 5′ sequence of miR-182) downstream of the firefly luciferase cDNA in the pRL-TK vector (Fig. 5A, bottom). We found that co-transfection of miR-182 mimics into 293T cells substantially decreased the luciferase activity of the wild-type reporter but barely affected that of the mutant reporter, suggesting that KLHL21 is a target of miR-182 (Fig. 5B). To corroborate this, we further examined the effect of miR-182 on endogenous *KLHL21* expression in A549 cells. Western blot confirmed that KLHL21 protein level was significantly reduced in miR-182 overexpressing A549 cells relative to NC control, while qPCR showed that the mRNA level was also significantly reduced (Fig. 5C). In contrast, inhibition of miR-182 by anti-miR-182 in A549-BM cells led to enhanced *KLHL21* expression (Fig. 5D). These results together support *KLHL21* as an authentic target of miR-182. To verify whether miR-182 promotes *IL-8* expression in NSCLC cells through targeting *KLHL21*, we constructed a *KLHL21* expression vector (p3xFlag-KLHL21), which lacks the *KLHL21* 3′UTR, for ectopic expression of Flag-KLHL21. Our qPCR and ELISA assays confirmed that restoration of *KLHL21* expression in miR-182-overexpressing A549 cells dramatically abrogated the effect of miR-182 on IL-8 expression and secretion (Fig. 5E, F). Collectively, these results support that miR-182 in NSCLC regulates IL-8 expression and secretion via the KLHL21:NF-κB regulatory axis.

### IL-8 activates osteoclastogenesis via STAT3 signaling

**Fig. 6:**
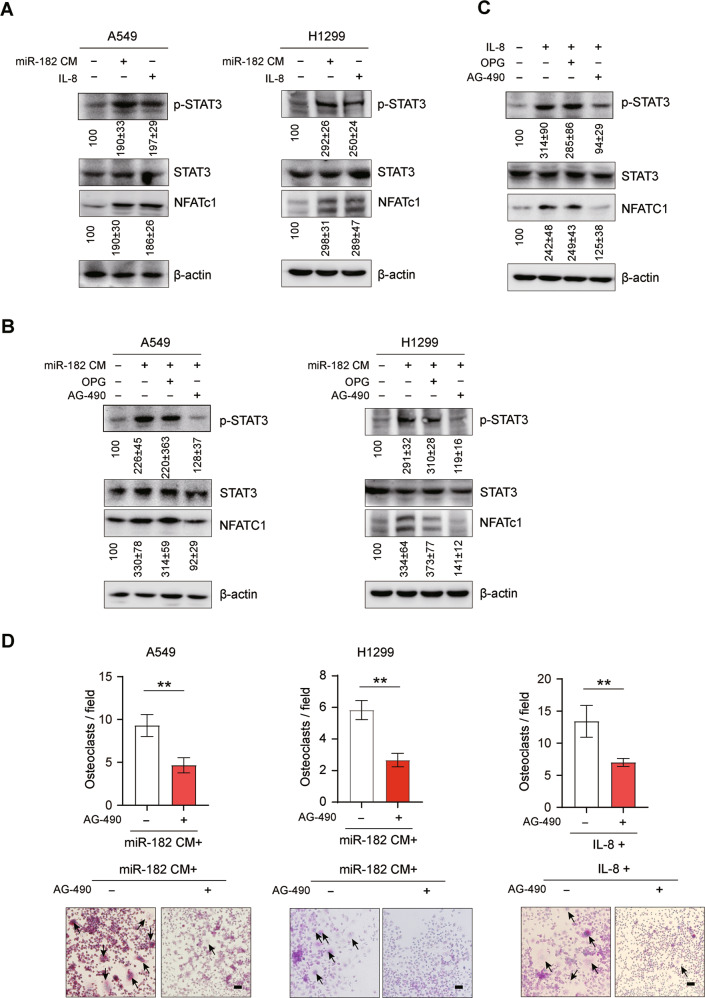
IL-8 activates osteoclastogenesis via STAT3 signaling. **A** Western blotting of the effects of miR-182-CM or IL-8 on STAT3 phosphorylation and NFATc1 expression in RAW264.7 cells. Quantification of blotting intensity is shown in parentheses (the one in control cells is set as 100% after normalization with β-actin). **B**, **C** Western blotting of the effects of JAK/STAT3 signaling inhibitor AG-490 and RANKL decoy receptor Osteoprotegerin (OPG) on STAT3 phosphorylation and NFATc1 expression in miR-182-CM (**B**) or IL-8 (**C**) -treated RAW264.7 cells, with quantification of blotting intensity shown in underneath (the one in control cells is set as 100% after normalization with β-actin). **D** TRAP staining analysis of the effect of AG-490 treatment on osteoclast differentiation of miR-182-CM or IL-8-treated RAW264.7 cells. Arrowheads indicate mature, multi-nucleated osteoclasts. Scale bars, 50 μm. The average values ± SEM of three separate experiments are plotted. **P < 0.01.

We further asked how IL-8 acts to facilitate NSCLC-induced osteoclastogenesis. To this end, we examined the effect of IL-8 on several well-documented signaling pathways involved in osteoclast differentiation, including STAT3, NF-κB, ERK and PI3K, in RAW264.7 cells [33–35]. We found that either miR-182-CM or recombinant IL-8 treatments primarily activated the STAT3 signaling but barely altered other tested signaling pathways (Figs. 6A and S6). In line with a previous study showing that STAT3 promotes osteoclast differentiation by upregulating the osteoclast differentiation key regulator NFATc1 [45], we found both miR-182-CM or IL-8 treatments significantly elevated NFATc1 protein expression in RAW264.7 cells (Fig. 6A). Consistent with our above results showing miR-182-CM promotes osteoclastogenesis in a RANKL-independent manner (Fig. 4A), OPG treatment little altered STAT3 phosphorylation and NFATc1 expression in miR-182-CM or IL-8-treated RAW264.7 cells (Fig. 6B, C). In sharp contrast, treatment with AG-490, a small molecular inhibitor for JAK/STAT3 signaling, dramatically abolished the stimulatory effect of miR-182-CM or IL-8 treatment on STAT3 phosphorylation and NFATc1 expression in RAW264.7 cells (Fig. 6B, C). Moreover, our ex vivo osteoclast differentiation assay showed that AG-490 markedly suppressed the stimulatory effect of miR-182-CM or IL-8 treatment on RAW264.7 cell differentiation (Fig. 6D), supporting that STAT3 signaling is responsible for miR-182-CM or IL-8-induced osteoclastogenesis. These results together suggest that miR-182-CM treatments promotes osteoclast differentiation via activating the STAT3 signaling pathway in osteoclast precursors cells.

### Systemic delivery of IL-8 neutralizing antibody inhibits the osteolytic bone metastasis of NSCLC cells in nude mice

**Fig. 7:**
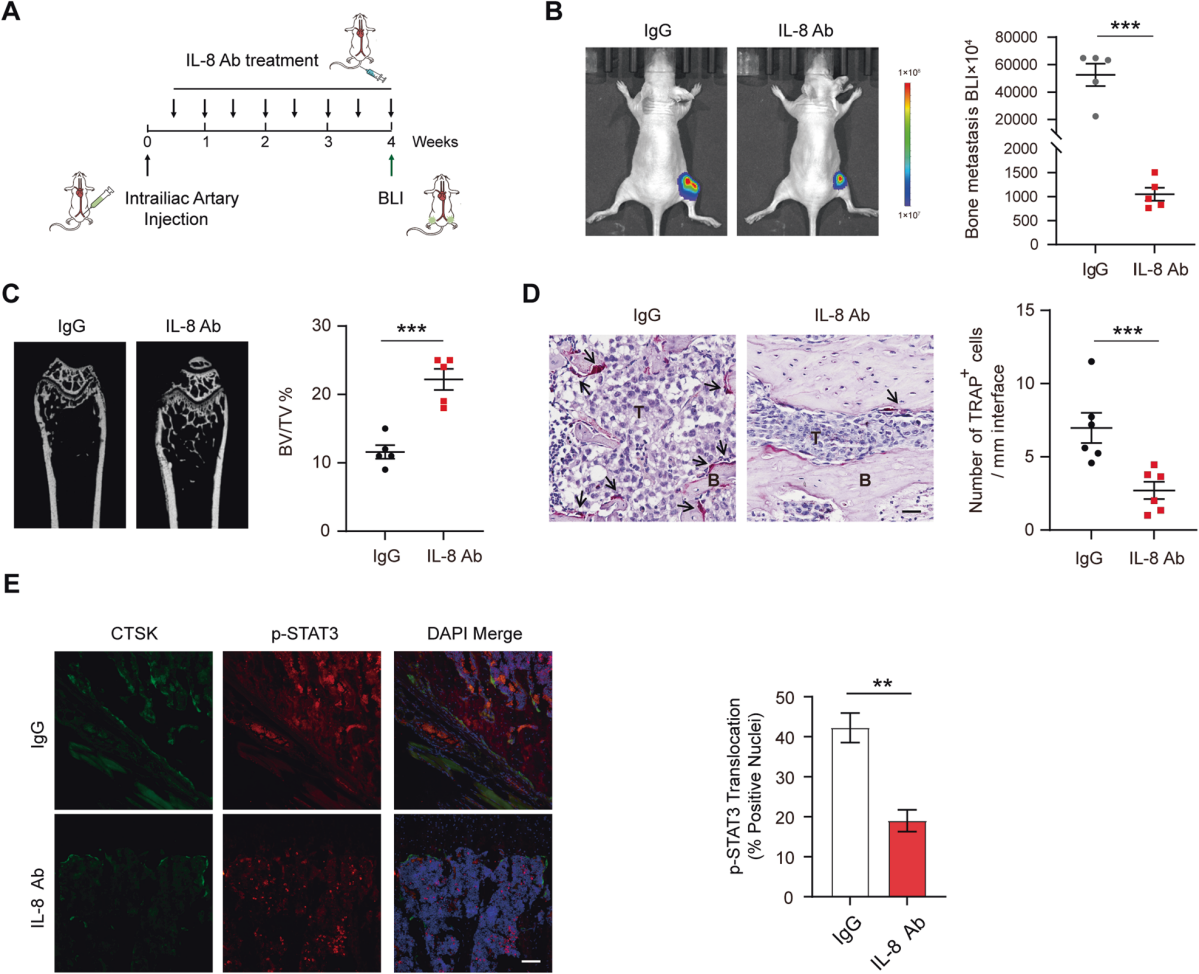
Systemic delivery of IL-8 neutralizing antibody overrides NSCLC bone metastasis in nude mice. **A** Schematic diagram illustrating the experimental design. The immunodeficient mice (5 for each group) were administered by IIA injection with miR-182-overexpressing A549 cells, followed by intravenous injection of IL-8 neutralizing antibody (0.1 mg/mouse) twice a week for 3 weeks. **B**–**D** IL-8 neutralizing antibody significantly inhibited bone metastasis formation in miR-182-overexpressing A549 cell-injected mice. At the treatment end (week 4), the bone-metastatic tumors were detected by BLI signals (**B**), bone damage was measured by micro-CT and quantified by BV/TV (**C**), and tumor-induced osteoclastogenesis was analyzed by TRAP staining, with TRAP-positive osteoclasts indicated by arrowheads (**D**). **E** Co-immunostaining of p-STAT3 (red) and the osteoclast marker CTSK (green) in distal femurs from two groups at the treatment end (week 4) (left). Quantification of p-STAT3^+^ osteoclasts in metastatic lesions (right). Scale bars, 50 μm. The average values ± SEM of three separate experiments are plotted. **P < 0.01; ***P < 0.001.

Would IL-8 be functionally required for miR-182-driven osteolytic bone metastasis of lung cancer in vivo? To address this question, we implanted the miR-182-expressing lentivirus-infected A549 cells into nude mice via intrailiac artery injection. 3 days post-implantation, IL-8 neutralizing antibody was delivered to the mice by intravenous injection, with twice a week for consecutive three weeks (Fig. 7A). Indeed, treatment with IL-8 neutralizing antibody led to a significant reduction of tumor burden in the hindlimb of mice, osteolytic bone lesion areas, and osteoclasts within bone-metastatic lesions in the hindlimb of mice (Fig. 7B–D). Moreover, our immunostaining showed a significant reduction of STAT3 phosphorylation in osteoclasts in metastatic niches from IL-8 neutralizing antibody-treated mice compared with IgG treatment controls (Fig. 7E; osteoclasts were indicated by the marker CTSK). These results together indicate that IL-8 is required for osteolytic bone metastasis of NSCLC cells in nude mice, supporting the therapeutic efficacy of IL-8 neutralizing antibody against osteolytic bone metastasis of lung cancer.

### The expression of miR-182 and IL-8 is correlated in human bone-metastatic lung cancer specimens

**Fig. 8:**
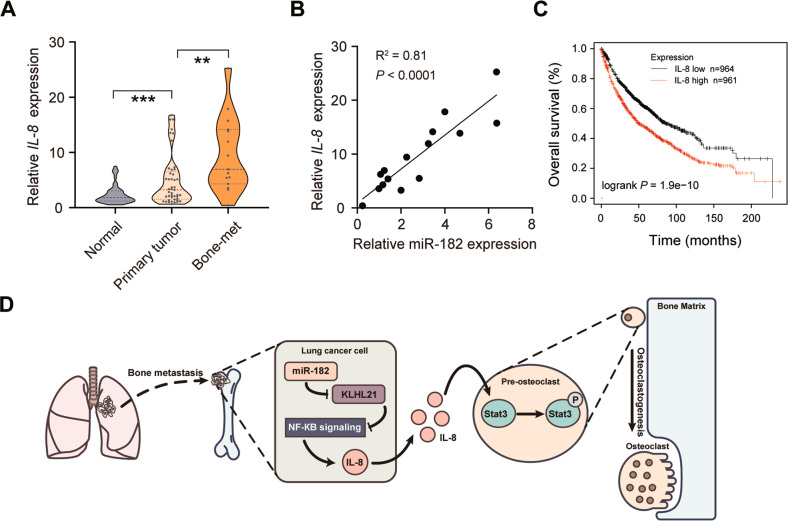
Comparison of miR-182 and IL-8 expression in bone-metastatic tumor specimens from NSCLC patients. **A** Quantification of the IL-8 level in primary NSCLC tumors (Primary tumor, *n* = 45), non-tumor lung tissues (Normal, *n* = 45), and bone metastatic tumors from NSCLC patients (Bone-met, *n* = 15) using qPCR. **B** Pearson’s correlation analysis of IL-8 and miR-182 levels in 15 bone-metastatic tumor specimens. **C** Comparison of the overall survival between the 1925 NSCLC patients with high or low levels of IL-8 by Kaplan–Meier analysis. Gene expression data and overall survival information were obtained from GEO, EGA and TCGA (CAARRAY, GSE14814, GSE19188, GSE29013, GSE30219, GSE31210, GSE3141, GSE31908, GSE37745, GSE43580, GSE4573, GSE50081, GSE8894, TCGA). **D** Schematic model showing that the miR-182/IL-8 regulatory axis plays an important role in regulating bone metastasis of lung cancer. The average values ± SEM of three separate experiments are plotted. **P < 0.01; ***P < 0.001.

To test whether our above findings in NSCLC and pre-osteoclast cells are clinically relevant, we examined the levels of *IL-8* in a cohort of human NSCLC primary tumors (*n* = 45), non-tumorous adjacent tissues (*n* = 45) and bone metastatic tumors (*n* = 15). Similar to miR-182 expression (Fig. 1E), *IL-8* expression was significantly upregulated in primary NSCLC tumors relative to adjacent normal tissues, and further upregulated in metastatic bone tumors (Fig. 8A). Importantly, we found a positive correlation between miR-182 and IL-8 levels in the bone metastatic tumors (Fig. 8B). Notably, by Kaplan–Meier analysis, we found that *IL-8* expression in tumors was inversely correlated with the overall survival in NSCLC patients (Fig. 8C). Collectively, these data suggest that the miR-182/IL-8 regulatory axis is involved in the interaction between tumor and bone stromal cells during the outgrowth of metastatic lung cancer cells, thereby damaging bone structures and resulting in lung cancer bone metastasis in the patients.

## Discussion

During bone metastasis, cytokine-mediated crosstalk between cancer cells and local bone cells may create a microenvironment to modulate bone homeostasis for favoring the colonization of cancer cells. For examples, bone-metastatic breast cells can produce high levels of bone-modulating factors, such as IL-1β and IL-11, to facilitate their spreading to the bone and subsequent metastatic outgrowth in the niche [11, 46, 47]. Also, both bone-metastatic myeloma and breast cancer cells are able to stimulate the production of IL-6 by bone marrow stromal cells, resulting in increased osteoclast differentiation and bone destruction [48, 49]. Moreover, IL-1β and IL-7R axis predominantly induces osteoblastic lesions and supports the skeletal colonization and metastatic progression of prostate cancer [50, 51]. However, whether and how cytokines or growth factors deposited in osseous lesions promote lung cancer bone metastasis remains largely unexplored. In the present study, we found that bone-metastatic NSCLC cell-produced IL-8 is functionally required for their osteolytic bone metastasis, and also showed that targeting IL-8 could be a new strategy to interrupt lung cancer bone metastasis.

Notably, IL-8 can be produced by various types of stromal cells in the tumor microenvironment, such as monocytes, endothelial cells, and lymphocytes. Multiple studies have shown that IL-8 is also overexpressed in a variety of cancer cell lines and functions as an autocrine and/or paracrine growth factor to facilitate tumor growth, invasion, metastasis, and angiogenesis [52–54]. Interestingly, our findings indicate that IL-8 secreted by bone-metastatic NSCLC cells acts to induce osteoclastogenesis independent of RANKL but dependent on JAK/STAT3 signaling. Our observation is echoed by a previous study reporting that osteoclast-specific *Stat3* deficiency causes impaired bone catabolism in mice [45], and also reminiscent of a recent study showing JAK/STAT3 as the major signaling pathway for interleukin actions in regulating osteoclast differentiation [55]. These findings together suggest that IL-8/STAT3 signaling is important for cancer cell-induced osteoclast differentiation in the metastatic niche.

MiR-182 is highly expressed in sensory tissues and organs, and its function is involved in the inner ear and retina development, as well as osteoclast and T cell differentiation [56]. Recent studies have shown that miR-182 exerts a regulatory effect on tumor occurrence, progression, and distant metastasis. In melanoma, dysregulation of miR-182 promotes tumor metastasis expressed by targeting *FoxO3* [25]. While TGF-β-induced miR-182 inhibited SMAD7 to promote EMT, invasion, and distant metastasis of breast cancer cells [26]. Despite that it remains controversial about the role of miR-182 in lung tumor occurrence and development [27, 28], our data indicate miR-182 as a critical regulator in NSCLC cells for bone metastasis and further demonstrate that miR-182 promotes NSCLC cell-induced osteolysis and metastatic burden by enhancing IL-8 secretion. Indeed, miR-182 has been shown to play a role in the TGF-β-induced bone metastasis of breast cancer [26] and TNF-α-mediated osteoclastogenesis [57]. Thus, these findings together suggest that upregulation of miR-182 in bone-metastatic cancer cells may represent a common mechanism linking inflammatory signaling and osteoclastogenesis in the metastatic niche.

In summary, our study identified the miR-182/IL-8/STAT3 axis as an important signaling pathway in regulating osteolytic metastasis of lung cancer. Importantly, these findings provide direct support for IL-8 as a therapeutic target for lung cancer patients with bone metastatic potential.

## Supplementary information

supplementary file

Original Data File

Reproducibility Checklist

## Data Availability

The data used to support the findings of this study are included within the article.

## References

[1] Sung H, Ferlay J, Siegel RL, Laversanne M, Soerjomataram I, Jemal A (2021). Global Cancer Statistics 2020: GLOBOCAN estimates of incidence and mortality worldwide for 36 cancers in 185 countries. CA Cancer J Clin.

[2] Ajona D, Ortiz-Espinosa S, Moreno H, Lozano T, Pajares MJ, Agorreta J (2017). A combined PD-1/C5a blockade synergistically protects against lung cancer growth and metastasis. Cancer discovery.

[3] Wang M, Wu Q, Zhang J, Qin G, Yang T, Liu Y (2021). Prognostic impacts of extracranial metastasis on non-small cell lung cancer with brain metastasis: a retrospective study based on surveillance, epidemiology, and end results database. Cancer Med.

[4] Chen YY, Wang PP, Fu Y, Li Q, Tian JF, Liu T (2021). Inferior outcome of bone metastasis in non-small-cell-lung-cancer patients treated with epidermal growth factor receptor inhibitors. J Bone Oncol.

[5] Zhang B, Li Y, Wu Q, Xie L, Barwick B, Fu C (2021). Acetylation of KLF5 maintains EMT and tumorigenicity to cause chemoresistant bone metastasis in prostate cancer. Nat Commun.

[6] Esposito M, Fang C, Cook KC, Park N, Wei Y, Spadazzi C (2021). TGF-β-induced DACT1 biomolecular condensates repress Wnt signalling to promote bone metastasis. Nat Cell Biol.

[7] Kfoury Y, Baryawno N, Severe N, Mei S, Gustafsson K, Hirz T (2021). Human prostate cancer bone metastases have an actionable immunosuppressive microenvironment. Cancer Cell.

[8] Bendre MS, Gaddy-Kurten D, Mon-Foote T, Akel NS, Skinner RA, Nicholas RW (2002). Expression of interleukin 8 and not parathyroid hormone-related protein by human breast cancer cells correlates with bone metastasis in vivo. Cancer Res.

[9] Sethi N, Dai X, Winter CG, Kang Y (2011). Tumor-derived JAGGED1 promotes osteolytic bone metastasis of breast cancer by engaging notch signaling in bone cells. Cancer Cell.

[10] Kang Y, Siegel PM, Shu W, Drobnjak M, Kakonen SM, Cordón-Cardo C (2003). A multigenic program mediating breast cancer metastasis to bone. Cancer Cell.

[11] Liang M, Ma Q, Ding N, Luo F, Bai Y, Kang F (2019). IL-11 is essential in promoting osteolysis in breast cancer bone metastasis via RANKL-independent activation of osteoclastogenesis. Cell Death Dis.

[12] Mulholland BS, Forwood MR, Morrison NA (2019). Monocyte chemoattractant protein-1 (MCP-1/CCL2) drives activation of bone remodelling and skeletal metastasis. Curr Osteoporos Rep.

[13] Wong SK, Mohamad NV, Giaze TR, Chin KY, Mohamed N, Ima-Nirwana S. Prostate cancer and bone metastases: the underlying mechanisms. Int J Mol Sci. 2019;20:2587. 10.3390/ijms20102587 PMC6567184 31137764

[14] Li X, Loberg R, Liao J, Ying C, Snyder LA, Pienta KJ (2009). A destructive cascade mediated by CCL2 facilitates prostate cancer growth in bone. Cancer Res.

[15] Furesi G, Rauner M, Hofbauer LC (2021). Emerging players in prostate cancer-bone niche communication. trends. Cancer..

[16] Wu S, Pan Y, Mao Y, Chen Y, He Y (2021). Current progress and mechanisms of bone metastasis in lung cancer: a narrative review. Transl Lung Cancer Res.

[17] Wang L, Zhang LF, Wu J, Xu SJ, Xu YY, Li D (2014). IL-1β-mediated repression of microRNA-101 is crucial for inflammation-promoted lung tumorigenesis. Cancer Res.

[18] Zhang LF, Lou JT, Lu MH, Gao C, Zhao S, Li B (2015). Suppression of miR-199a maturation by HuR is crucial for hypoxia-induced glycolytic switch in hepatocellular carcinoma. EMBO J.

[19] Cai WL, Huang WD, Li B, Chen TR, Li ZX, Zhao CL (2018). microRNA-124 inhibits bone metastasis of breast cancer by repressing Interleukin-11. Mol Cancer.

[20] Browne G, Taipaleenmäki H, Stein GS, Stein JL, Lian JB (2014). MicroRNAs in the control of metastatic bone disease. Trends Endocrinol Metab.

[21] Bellavia D, Salamanna F, Raimondi L, De Luca A, Carina V, Costa V (2019). Deregulated miRNAs in osteoporosis: effects in bone metastasis. Cell Mol Life Sci.

[22] Mann M, Barad O, Agami R, Geiger B, Hornstein E (2010). miRNA-based mechanism for the commitment of multipotent progenitors to a single cellular fate. Proc Natl Acad Sci USA.

[23] Ell B, Mercatali L, Ibrahim T, Campbell N, Schwarzenbach H, Pantel K (2013). Tumor-induced osteoclast miRNA changes as regulators and biomarkers of osteolytic bone metastasis. Cancer Cell.

[24] Wang M, Zhao M, Guo Q, Lou J, Wang L (2021). Non-small cell lung cancer cell-derived exosomal miR-17-5p promotes osteoclast differentiation by targeting PTEN. Exp Cell Res.

[25] Segura MF, Hanniford D, Menendez S, Reavie L, Zou X, Alvarez-Diaz S (2009). Aberrant miR-182 expression promotes melanoma metastasis by repressing FOXO3 and microphthalmia-associated transcription factor. Proc Natl Acad Sci USA.

[26] Yu J, Lei R, Zhuang X, Li X, Li G, Lev S (2016). MicroRNA-182 targets SMAD7 to potentiate TGFbeta-induced epithelial-mesenchymal transition and metastasis of cancer cells. Nat Commun.

[27] Seidl C, Panzitt K, Bertsch A, Brcic L, Schein S, Mack M (2020). MicroRNA-182-5p regulates hedgehog signaling pathway and chemosensitivity of cisplatin-resistant lung adenocarcinoma cells via targeting GLI2. Cancer Lett.

[28] Li Y, Zhang H, Gong H, Yuan Y, Li Y, Wang C (2018). miR-182 suppresses invadopodia formation and metastasis in non-small cell lung cancer by targeting cortactin gene. J Exp Clin Cancer Res.

[29] Mei ZZ, Chen XY, Hu SW, Wang N, Ou XL, Wang J (2016). Kelch-like protein 21 (KLHL21) targets IκB kinase-β to regulate nuclear factor κ-light chain enhancer of activated B cells (NF-κB) signaling negatively. J Biol Chem.

[30] Jiang S, Zhang LF, Zhang HW, Hu S, Lu MH, Liang S (2012). A novel miR-155/miR-143 cascade controls glycolysis by regulating hexokinase 2 in breast cancer cells. EMBO J.

[31] Li F, Yuan P, Rao M, Jin CH, Tang W, Rong YF (2020). piRNA-independent function of PIWIL1 as a co-activator for anaphase promoting complex/cyclosome to drive pancreatic cancer metastasis. Nat Cell Biol.

[32] Minn AJ, Gupta GP, Siegel PM, Bos PD, Shu W, Giri DD (2005). Genes that mediate breast cancer metastasis to lung. Nature..

[33] Brady JJ, Chuang CH, Greenside PG, Rogers ZN, Murray CW, Caswell DR (2016). An Arntl2-driven secretome enables lung adenocarcinoma metastatic self-sufficiency. Cancer Cell.

[34] Zhuang X, Zhang H, Li X, Li X, Cong M, Peng F (2017). Differential effects on lung and bone metastasis of breast cancer by Wnt signalling inhibitor DKK1. Nat Cell Biol.

[35] Lee CW, Ren YJ, Marella M, Wang M, Hartke J, Couto SS (2020). Multiplex immunofluorescence staining and image analysis assay for diffuse large B cell lymphoma. J Immunol Methods.

[36] Kang Y (2016). Imaging TGFβ signaling in mouse models of cancer metastasis. Methods Mol Biol.

[37] Wang H, Yu C, Gao X, Welte T, Muscarella AM, Tian L (2015). The osteogenic niche promotes early-stage bone colonization of disseminated breast cancer cells. Cancer Cell.

[38] Yu C, Wang H, Muscarella A, Goldstein A, Zeng HC, Bae Y, et al. Intra-iliac artery injection for efficient and selective modeling of microscopic bone metastasis. J Vis Exp. 2016;26:53982. 10.3791/53982 PMC5092070 27768029

[39] Chen H, Xu L, Wang L (2019). Expression of miR-182 and Foxo3a in patients with bladder cancer correlate with prognosis. Int J Clin Exp Pathol.

[40] Udagawa N, Koide M, Nakamura M, Nakamichi Y, Yamashita T, Uehara S (2021). Osteoclast differentiation by RANKL and OPG signaling pathways. J Bone Miner Metab.

[41] Krek A, Grün D, Poy MN, Wolf R, Rosenberg L, Epstein EJ (2005). Combinatorial microRNA target predictions. Nat Genet.

[42] Lewis BP, Burge CB, Bartel DP (2005). Conserved seed pairing, often flanked by adenosines, indicates that thousands of human genes are microRNA targets. Cell..

[43] Sankpal NV, Fleming TP, Gillanders WE (2013). EpCAM modulates NF-κB signaling and interleukin-8 expression in breast cancer. Mol Cancer Res.

[44] Wang S, Liu Z, Wang L, Zhang X (2009). NF-kappaB signaling pathway, inflammation and colorectal cancer. Cell Mol Immunol.

[45] Yang Y, Chung MR, Zhou S, Gong X, Xu H, Hong Y (2019). STAT3 controls osteoclast differentiation and bone homeostasis by regulating NFATc1 transcription. J Biol Chem.

[46] Nutter F, Holen I, Brown HK, Cross SS, Evans CA, Walker M (2014). Different molecular profiles are associated with breast cancer cell homing compared with colonisation of bone: evidence using a novel bone-seeking cell line. Endocr Relat Cancer.

[47] Lee ZH, Kim HH (2003). Signal transduction by receptor activator of nuclear factor kappa B in osteoclasts. Biochem Biophys Res Commun.

[48] Krishnan V, Shuman LA, Sosnoski DM, Dhurjati R, Vogler EA, Mastro AM (2011). Dynamic interaction between breast cancer cells and osteoblastic tissue: comparison of two- and three-dimensional cultures. J Cell Physiol.

[49] De S, Chen J, Narizhneva NV, Heston W, Brainard J, Sage EH (2003). Molecular pathway for cancer metastasis to bone. J Biol Chem.

[50] Liu Q, Russell MR, Shahriari K, Jernigan DL, Lioni MI, Garcia FU (2013). Interleukin-1β promotes skeletal colonization and progression of metastatic prostate cancer cells with neuroendocrine features. Cancer Res.

[51] Seol MA, Kim JH, Oh K, Kim G, Seo MW, Shin YK (2019). Interleukin-7 contributes to the invasiveness of prostate cancer cells by promoting epithelial-mesenchymal transition. Sci Rep.

[52] Ogawa R, Yamamoto T, Hirai H, Hanada K, Kiyasu Y, Nishikawa G (2019). Loss of SMAD4 promotes colorectal cancer progression by recruiting tumor-associated neutrophils via the CXCL1/8-CXCR2 Axis. Clin Cancer Res.

[53] Timani KA, Győrffy B, Liu Y, Mohammad KS, He JJ (2018). Tip110/SART3 regulates IL-8 expression and predicts the clinical outcomes in melanoma. Mol Cancer.

[54] Lesage J, Suarez-Carmona M, Neyrinck-Leglantier D, Grelet S, Blacher S, Hunziker W (2017). Zonula occludens-1/NF-κB/CXCL8: a new regulatory axis for tumor angiogenesis. FASEB J.

[55] Sanpaolo ER, Rotondo C, Cici D, Corrado A, Cantatore FP (2020). JAK/STAT pathway and molecular mechanism in bone remodeling. Mol Biol Rep.

[56] Wei Q, Lei R, Hu G (2015). Roles of miR-182 in sensory organ development and cancer. Thorac Cancer.

[57] Miller CH, Smith SM, Elguindy M, Zhang T, Xiang JZ, Hu X (2016). RBP-J-regulated miR-182 promotes TNF-alpha-induced osteoclastogenesis. J Immunol.

