# Supplementary material for: A novel microRNA-182/Interleukin-8 regulatory axis controls osteolytic bone metastasis of lung cancer
Source: Cell Death Dis. 2023 May 1;14(5):298. doi: 10.1038/s41419-023-05819-8 (PMC10151336; doi:10.1038/s41419-023-05819-8)
Supplement: Supplementary file 1 — supplementary file [file 41419_2023_5819_MOESM1_ESM.docx]

Supplementary Materials for

**A novel microRNA-182/Interleukin-8 regulatory axis controls osteolytic bone metastasis of lung cancer**

Ming-Na Zhao, Ling-Fei Zhang, Zhen Sun, Li-hua Qiao, Tao Yang, Yi-Zhe Ren, Xian-Zhou Zhang, Lei Wu, Wen-Li Qian, Qiao-mei Guo, Wan-Xing Xu, Xue-Qing Wang, Fei Wu, Lin Wang, Yutong Gu^*^, Mo-Fang Liu^*^, Jia-Tao Lou^*^

*Corresponding authors. Email: Jia-Tao Lou, loujiatao@sjtu.edu.cn (J.-T.L.); Mo-Fang Liu, [mfliu@sibcb.ac.cn](mailto:mfliu@sibcb.ac.cn) (M.-F.L.); Yutong Gu, [gu.yutong2@zs-hospital.sh.cn](mailto:gu.yutong2@zs-hospital.sh.cn) (Y.G.)

**This PDF file includes:**

Figs. S1 to S6

Table S1

**Supplementary Figures**


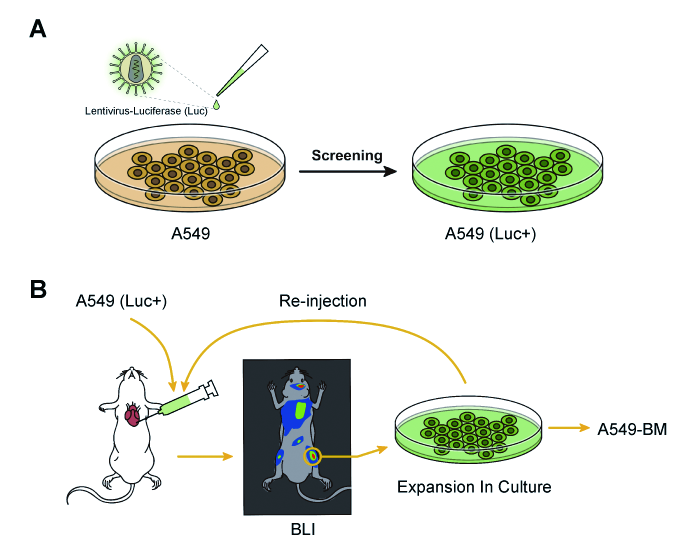


**Supplementary Figure 1. Generation of bone-metastatic A549 subline using a murine model of bone metastasis.** (A) Schematic diagram illustrating the experimental design for generating A549 subline stably expressing a luciferase reporter (referred to as A549-luc). A549 cells were infected with lentiviral vector pLvx-luc for expressing luciferase reporter and puro resistance genes, and the subline stably expressing luciferase was selected with purinomycin treatment. (B) Schematic diagram illustrating the experimental design for generating bone-metastatic A549 subline (referred to as A549-BM). A549-luc cells were administered into the left ventricle of the mice. 4 weeks later, A549-luc cells in bone tissue were isolated via BLI guiding, and injected into the mice again. After 3 cycles, the obtained subline became significantly prone to bone metastasis and was thus termed as A549-BM subline.


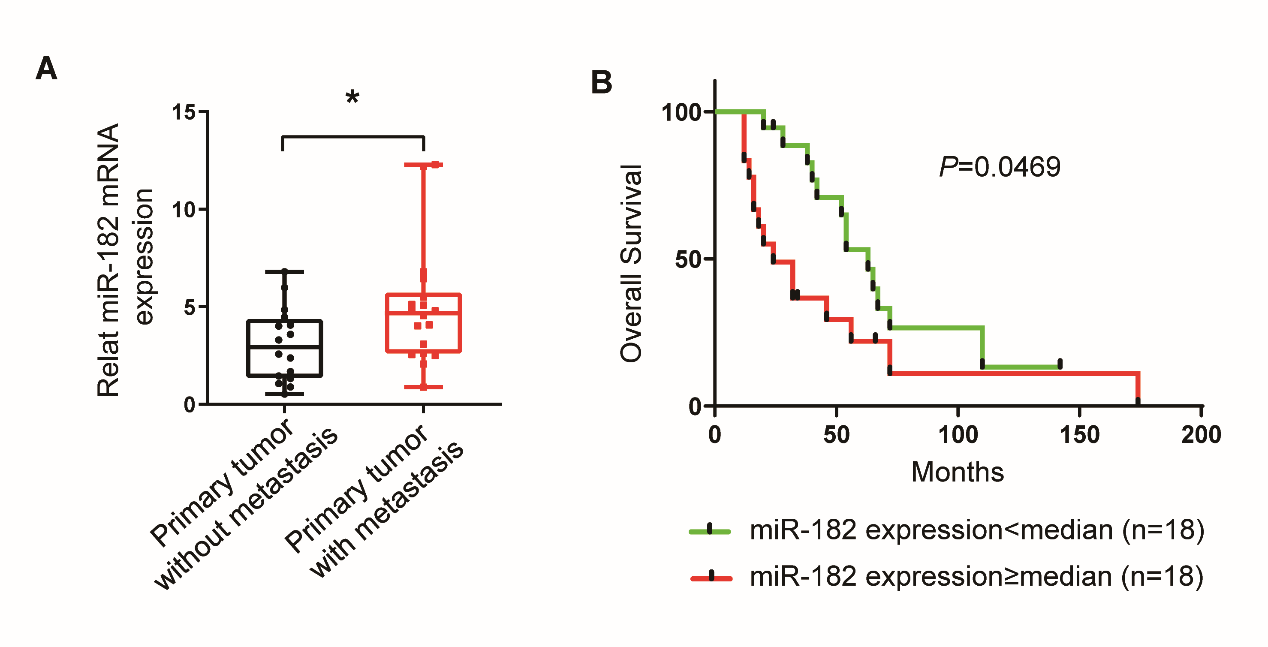


**Supplementary Figure 2.** **miR-182 expression is significantly elevated in metastatic NSCLC patients.** (A) qPCR assay of miR-182 level in primary tumors from metastatic (n=18) and non-metastatic NSCLC patients (n=18). (B) Comparison of the overall survival between the NSCLC patients with a higher (n=18) or lower level of miR-182 (n=18) by Kaplan-Meier analysis. The average values ± SEM of three separate experiments are plotted. *, *P* < 0.05.


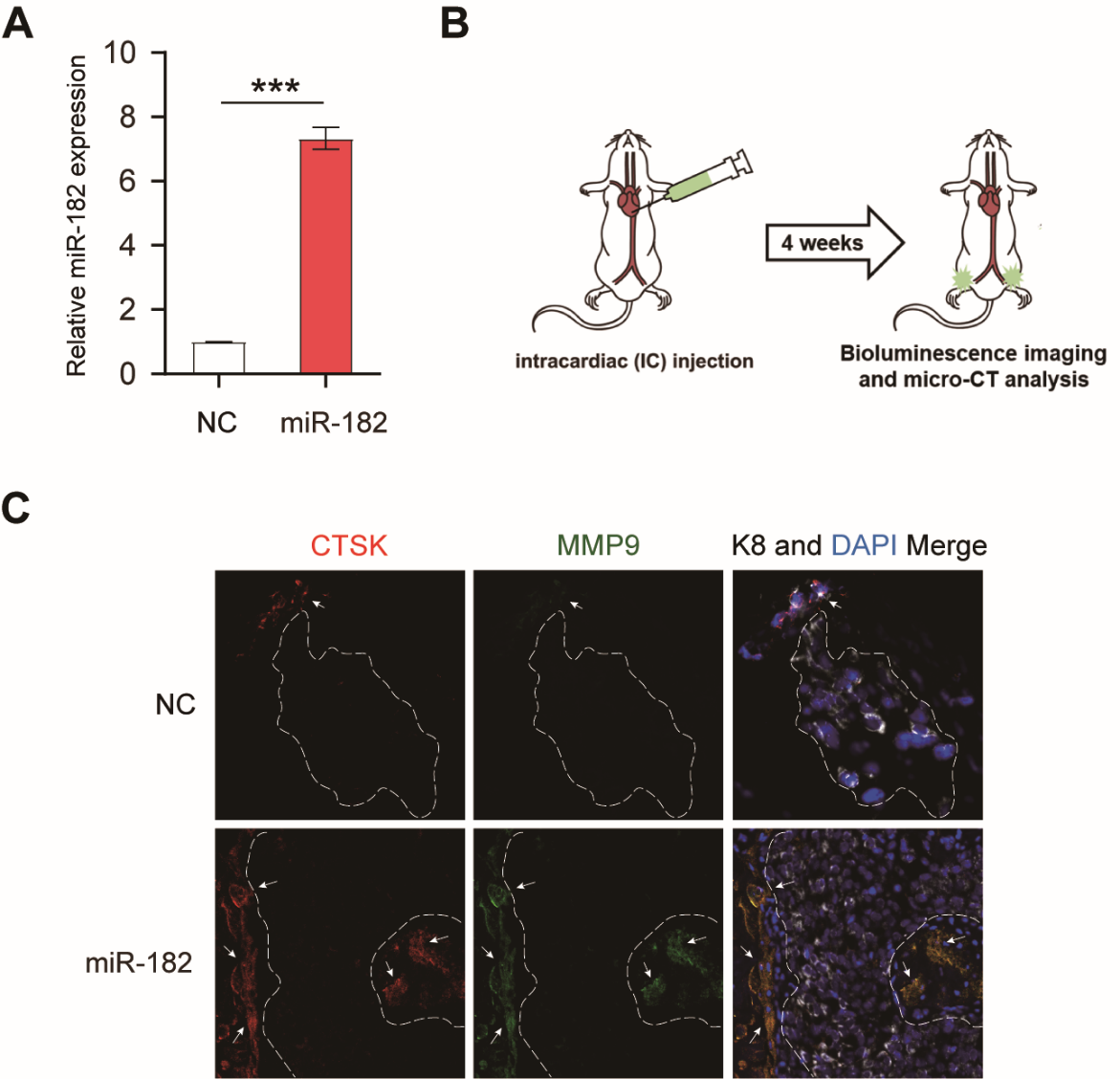


**Supplementary Figure 3. miR-182 promotes lung cancer bone metastasis in mice.** (A) qPCR quantification of miR-182 expression in miR-182 expression or negative control (NC) lentivirus-infected A549 cells. Expression of miR-182 was normalized against an endogenous control U6. (B) Schematic diagram illustrating the experimental procedure. The mice were administered intracardially with miR-182 expression or NC lentivirus-infected A549 cells (5 for each group). 4 weeks later, BLI and micro-CT were carried out to assess osteolysis as well as the growth of metastatic tumors. (C) Multiplex immunofluorescence staining analyses of CTSK (Red), MMP9 (Green), and K8 (White) for osteoclast formation at the boundary between bone and metastatic tumor in NC or miR-182 overexpressing A549 cell-injected mice. Arrowheads indicate the cells that showed both CTSK and MMP9 positive. The average values ± SEM of three separate experiments are plotted. ***, *P* < 0.001.

**
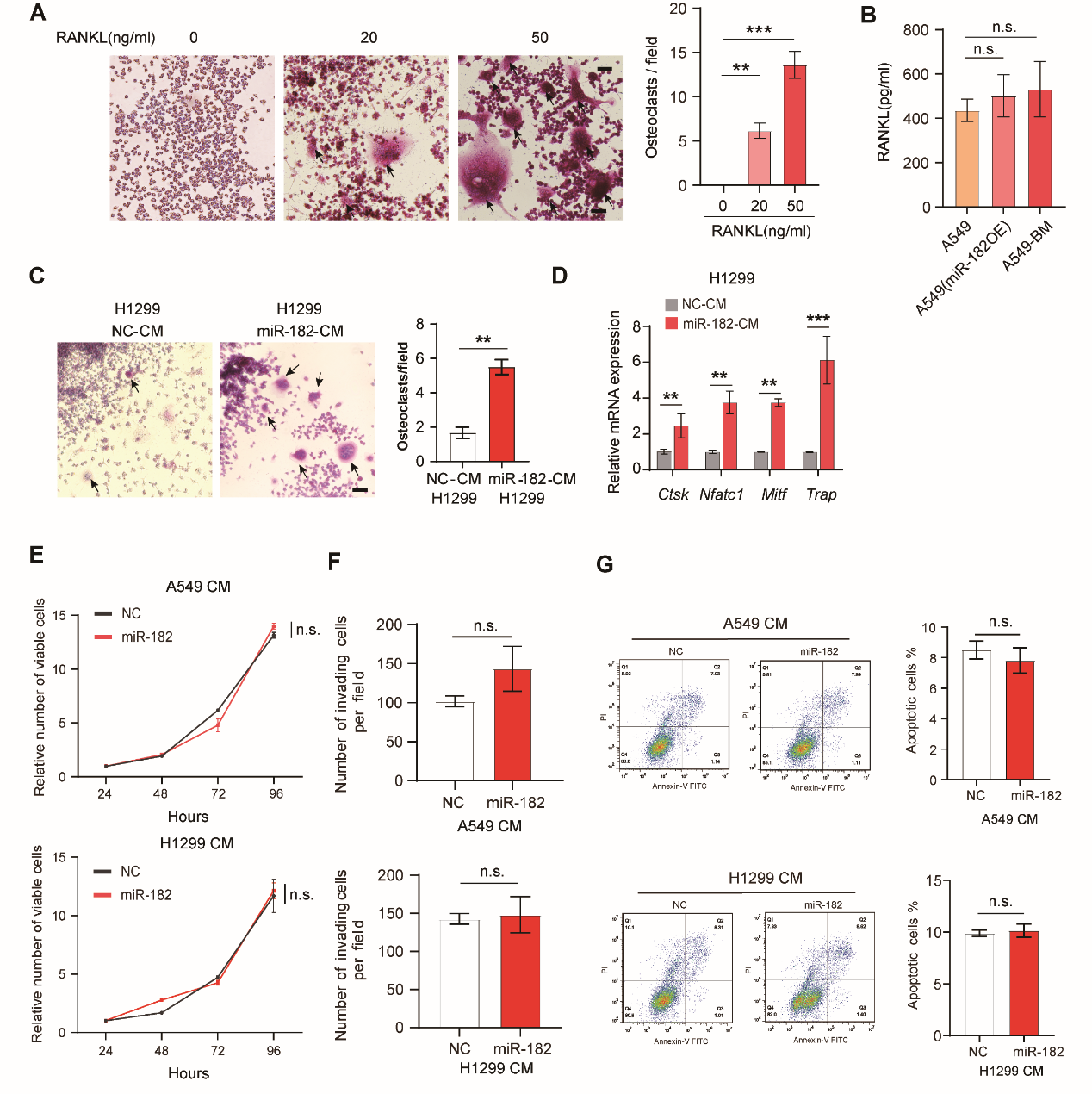
Supplementary Figure 4. Conditioned media (CM) from miR-182-upregulated NSCLC cells promotes osteoclast differentiation but barely alters cell proliferation, migration and apoptosis in RAW264.7 cells.** (A) TRAP staining analysis of osteoclast differentiation of RAW264.7 cells treated with indicated amount of RANKL. Left, representative TRAP staining images, with the mature, multi-nucleated osteoclasts indicated by arrowheads. Scale bars, 50 μm. Right, quantification of osteoclasts per field under indicated treatment. (B) ELISA analysis of RANKL level in the CM collected from indicated cells. (C) TRAP staining analysis of the osteoclast differentiation of RAW264.7 cells after cultured in the CM from NC or miR-182 overexpressing H1299 cells. Left, representative TRAP staining images, with the mature, multi-nucleated osteoclasts indicated by arrowheads. Scale bars, 50 μm. Right, quantification of osteoclasts per field under indicated treatment. (D) qPCR analysis of the effect of indicated CM on the expression of osteoclastogenesis-related genes in RAW264.7 cells. (E) MTT analysis of the effect of indicated CM on RAW264.7 cell proliferation. (F) Transwell analysis of the effect of indicated CM on RAW264.7 cell migration. (G) Annexin V staining analysis of the effect of indicated CM on RAW264.7 cell apoptosis. The average values ± SEM of three separate experiments are plotted. n.s., not significant; **, *P* < 0.01 and ***, *P* < 0.001.


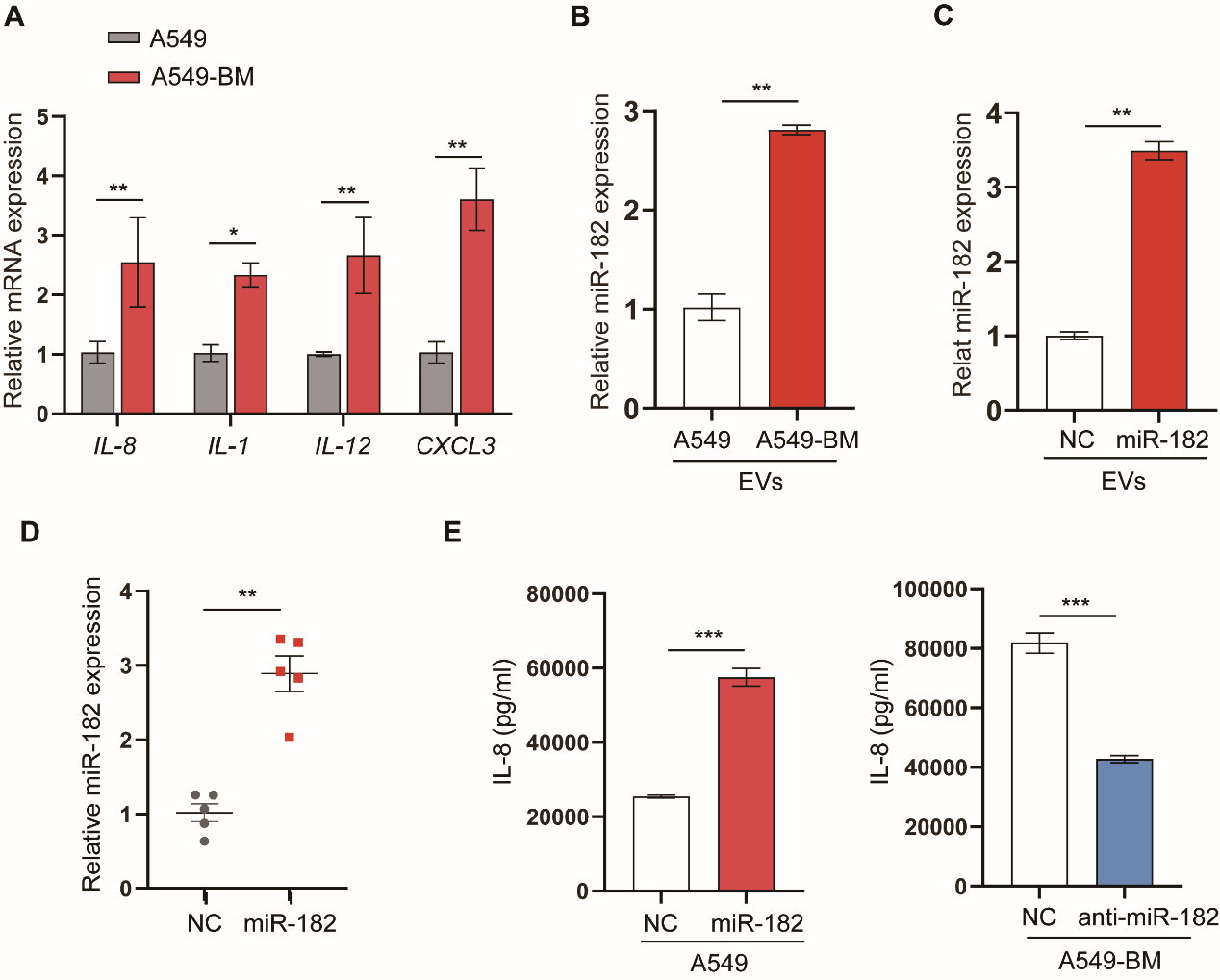


**Supplementary Figure 5. miR-182 upregulation promotes the expression of IL-8, IL-1, IL-12, and CXCL3 in NSCLC cells.** (A) qPCR quantification of IL-8, IL-1, IL-12, and CXCL3 expression in A549-BM cells and A549 cells. (B-D) qPCR analysis of miR-182 level in the EVs from A549-BM cells (B), miR-182-overexpressing A549 cells (C) or the blood of mice injected with miR-182-overexpressing A549 cells (D) compared with the respective controls. (E) ELISA analysis of IL-8 level in the CM from miR-182 expression or NC lentivirus-infected A549 cells. The average values ± SEM of three separate experiments are plotted. *, *P* < 0.05; **, *P* < 0.01 and ***, *P* < 0.001.


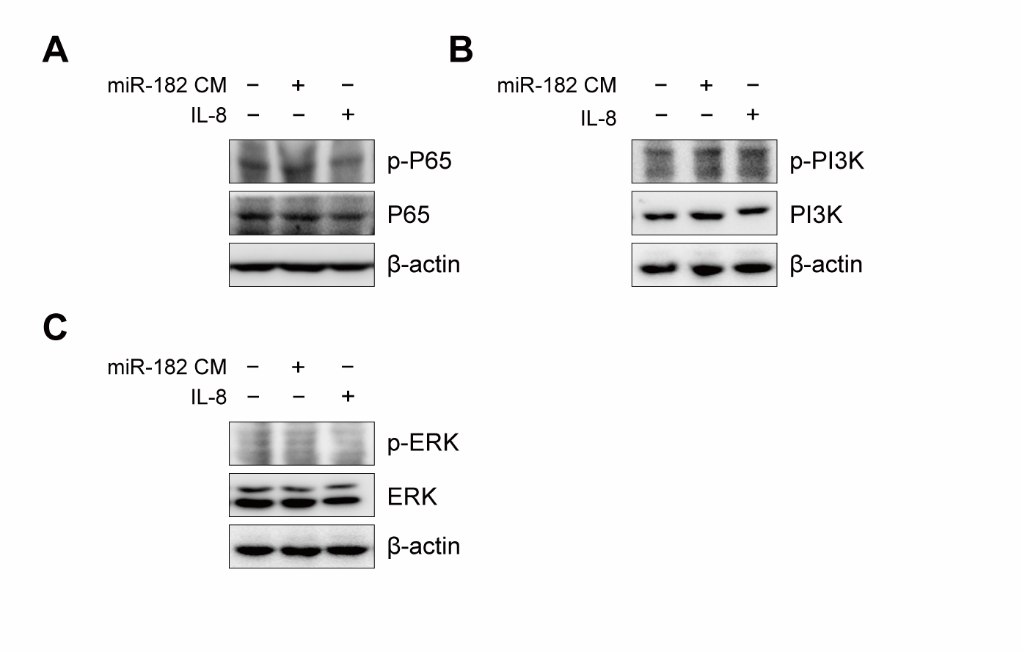


**Supplementary Figure 6. miR-182-CM or IL-8 treatments litter alter NF-κB, ERK, and PI3K signaling pathways in RAW264.7 cells.** Western blotting assay of the phosphorylation of P65 (A), ERK (B), and PI3K (C) in miR-182-CM or IL-8-treated RAW264.7 cells. Results shown are representative of three independent experiments.

**Supplementary Table S1.** Sequences of primers used in this study

| Genes | Forward (5′-3′) | Reverse (5′-3′) |
| --- | --- | --- |
| *Ctsk* | GAAGAAGACTCACCAGAAGCAG | TCCAGGTTATGGGCAGAGATT |
| *Trap* | GCTGGAAACCATGATCACCT | GAGTTGCCACACAGCATCAC |
| *Nfatc1* | GACCCGGAGTTCGACTTCG | TGACACTAGGGGACACATAACTG |
| *Mitf* | AGACCTGACATGTACGACAAC | TATGCAGGGCTACTGATAAG |
| *β-actin*  (mouse) | CGCCACCAGTTCGCCATGGA | TACAGCCCGGGGAGCATCGT |
| *β-actin*  (human) | ATCAAGATCATTGCTCCTCCTGAG | CTGCTTGCTGATCCACATCTG |
| *IL-8* | CACCTCAAGAACATCCAGAGCT | CAAGCAGAACTGAACTACCATCG |
| *IL-1* | CCATCCAACCCAGATCAGCA | GTTTCTGGCAACTCCTTCAGC |
| *IL-12* | TGCCTTCACCACTCCCAAAACC | TGCCTTCACCACTCCCAAAACC |
| *CXCL3* | TCCCCCATGGTTCAGAAAATC | GGTGCTCCCCTTGTTCAGTATCT |
| miR-182 | ACACTCCAGCTGGGTTTGGCAATGGTAGAACT | CTCAACTGGTGTCGTGGAGTCGG |
