# Supplementary material for: A novel microRNA-182/Interleukin-8 regulatory axis controls osteolytic bone metastasis of lung cancer
Source: Cell Death Dis. 2023 May 1;14(5):298. doi: 10.1038/s41419-023-05819-8 (PMC10151336; doi:10.1038/s41419-023-05819-8)

Figure 4E

A549 CM

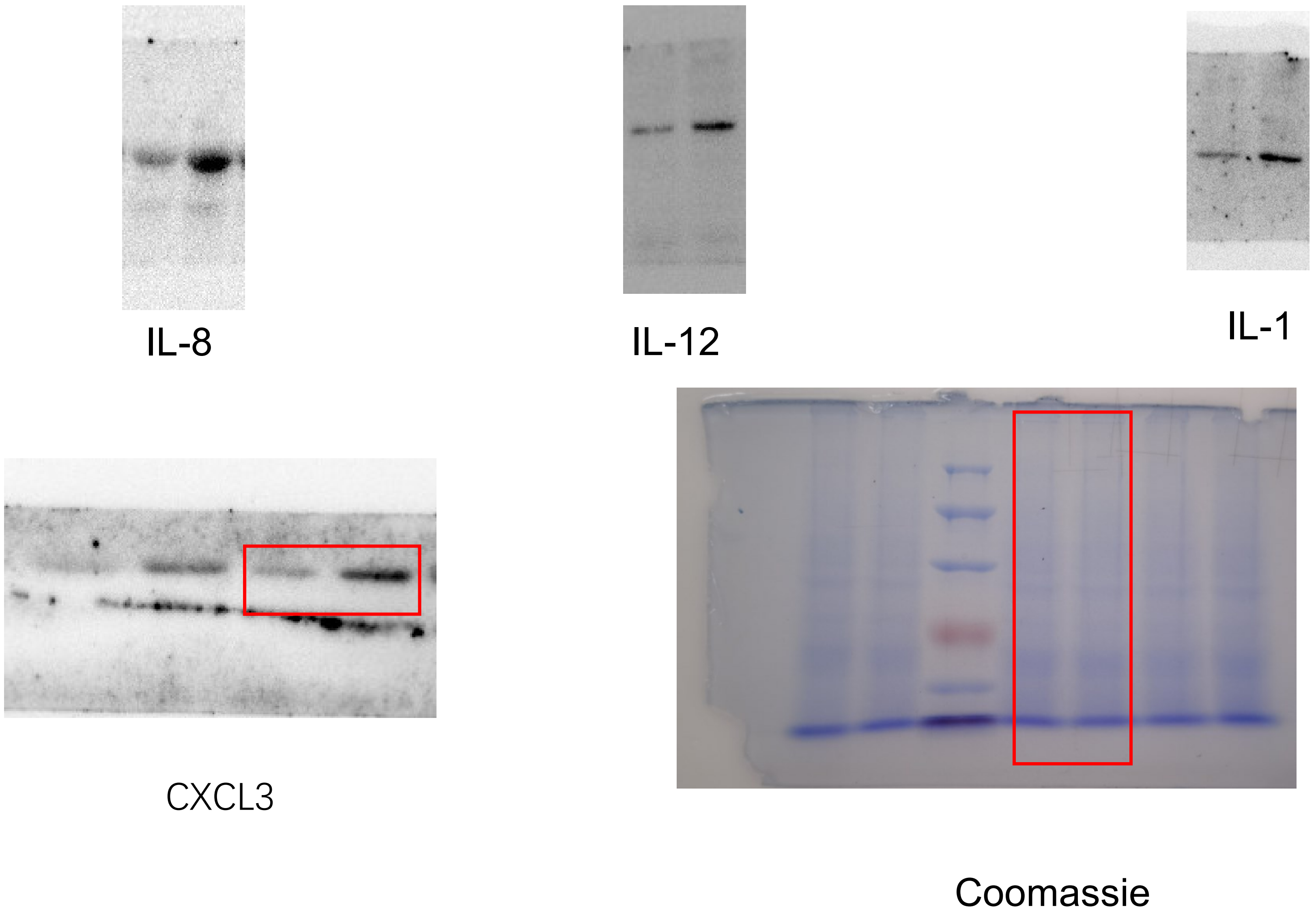

H1299 CM

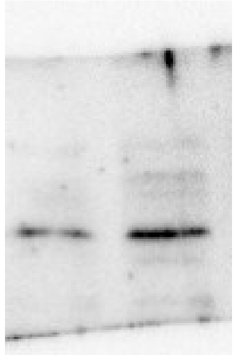

IL-8

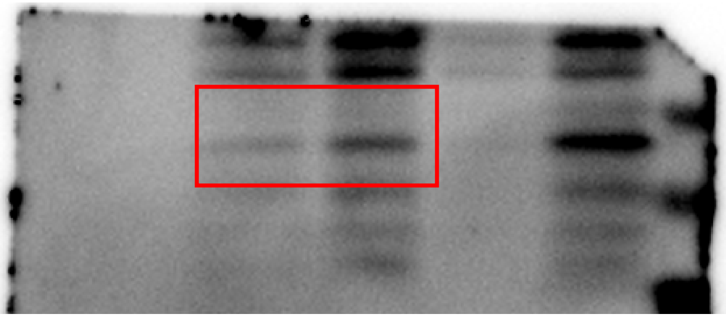

IL-1

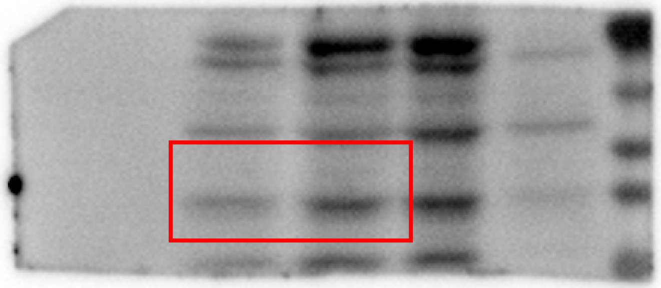

IL-12

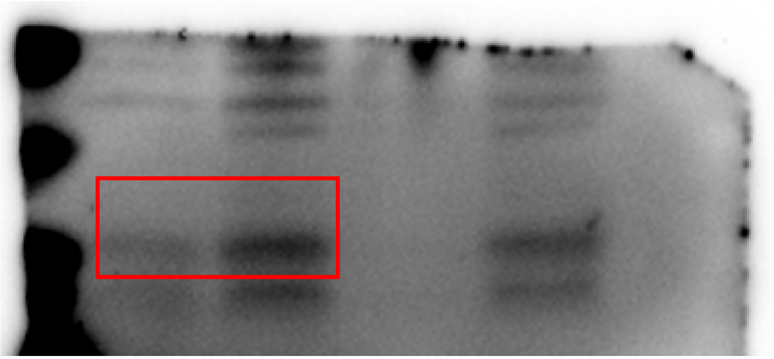

CXCL3

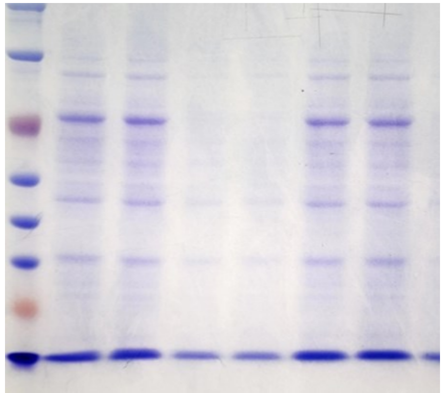

Coomassie

Figure 5C

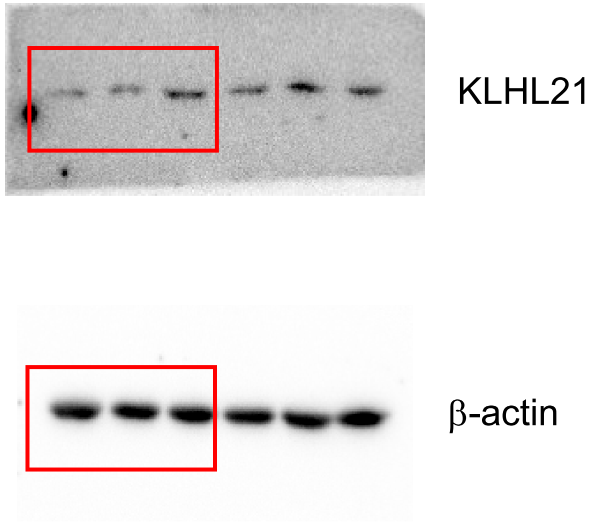

Figure 5D

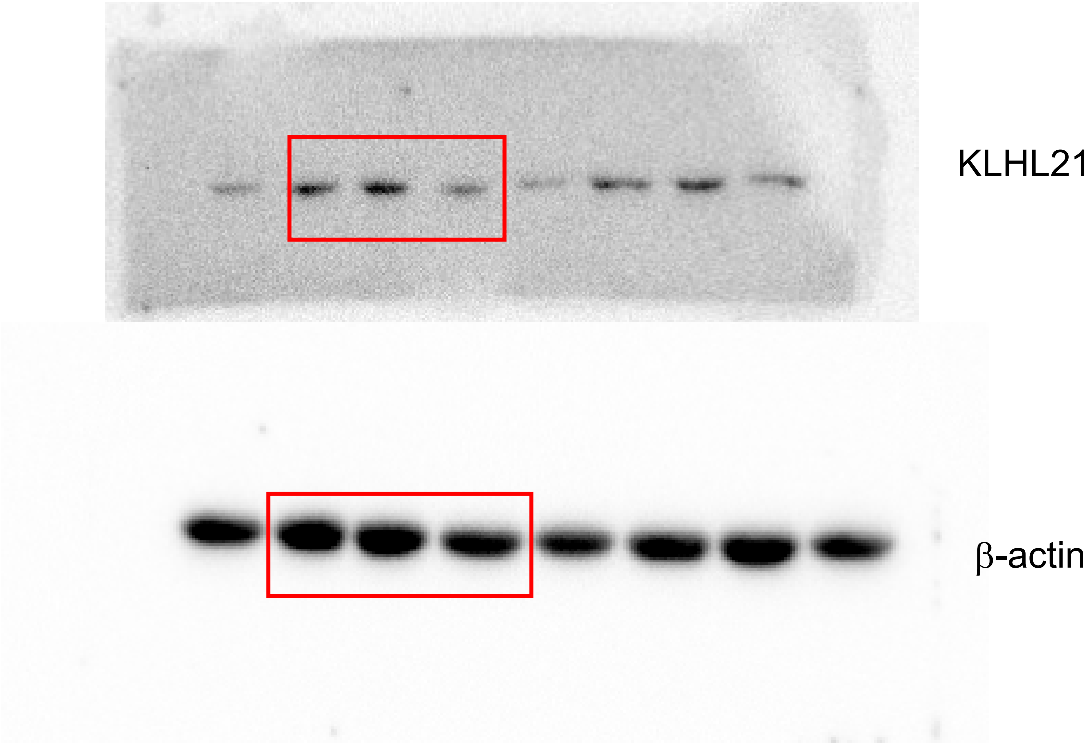

Figure 6A

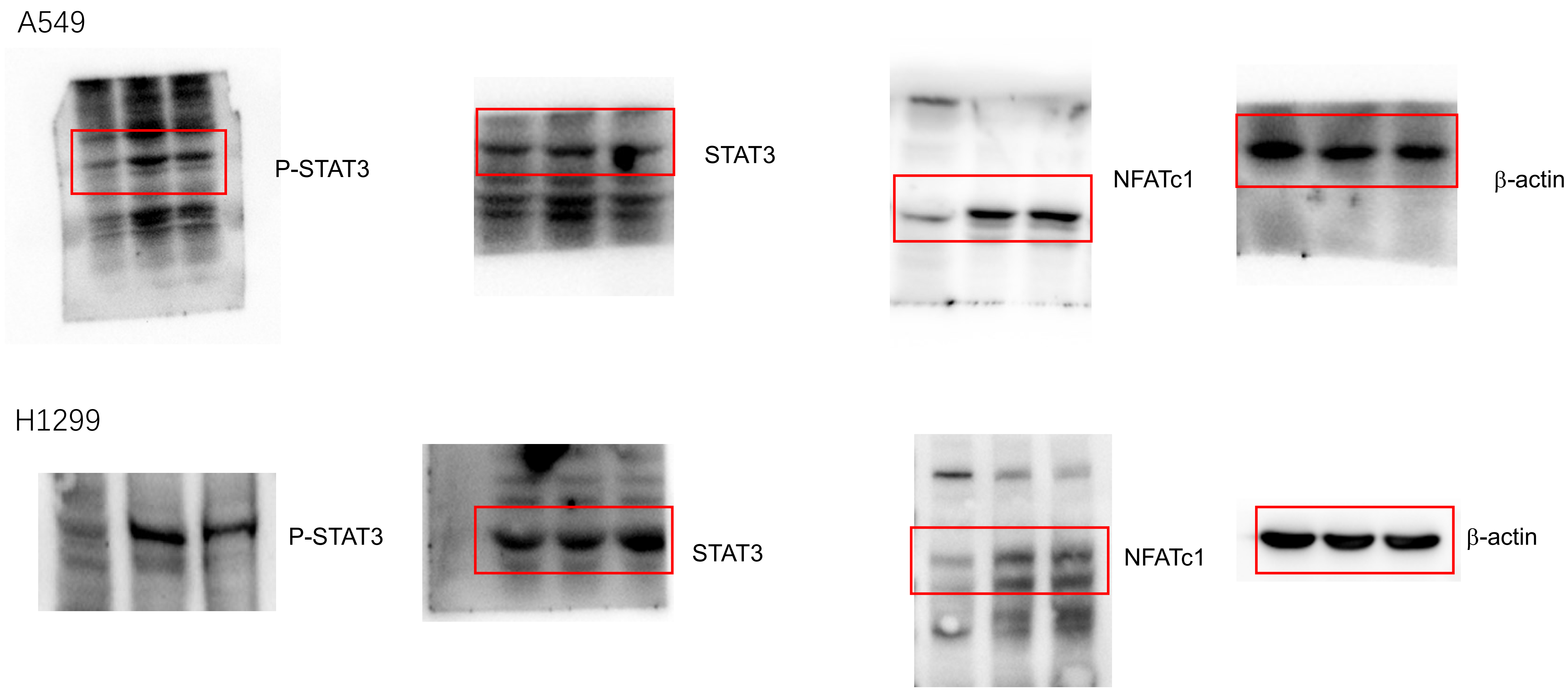
A549
P-STAT3
STAT3
NFATc1
β-actin
H1299
P-STAT3
STAT3
NFATc1
β-actin

Figure 6B

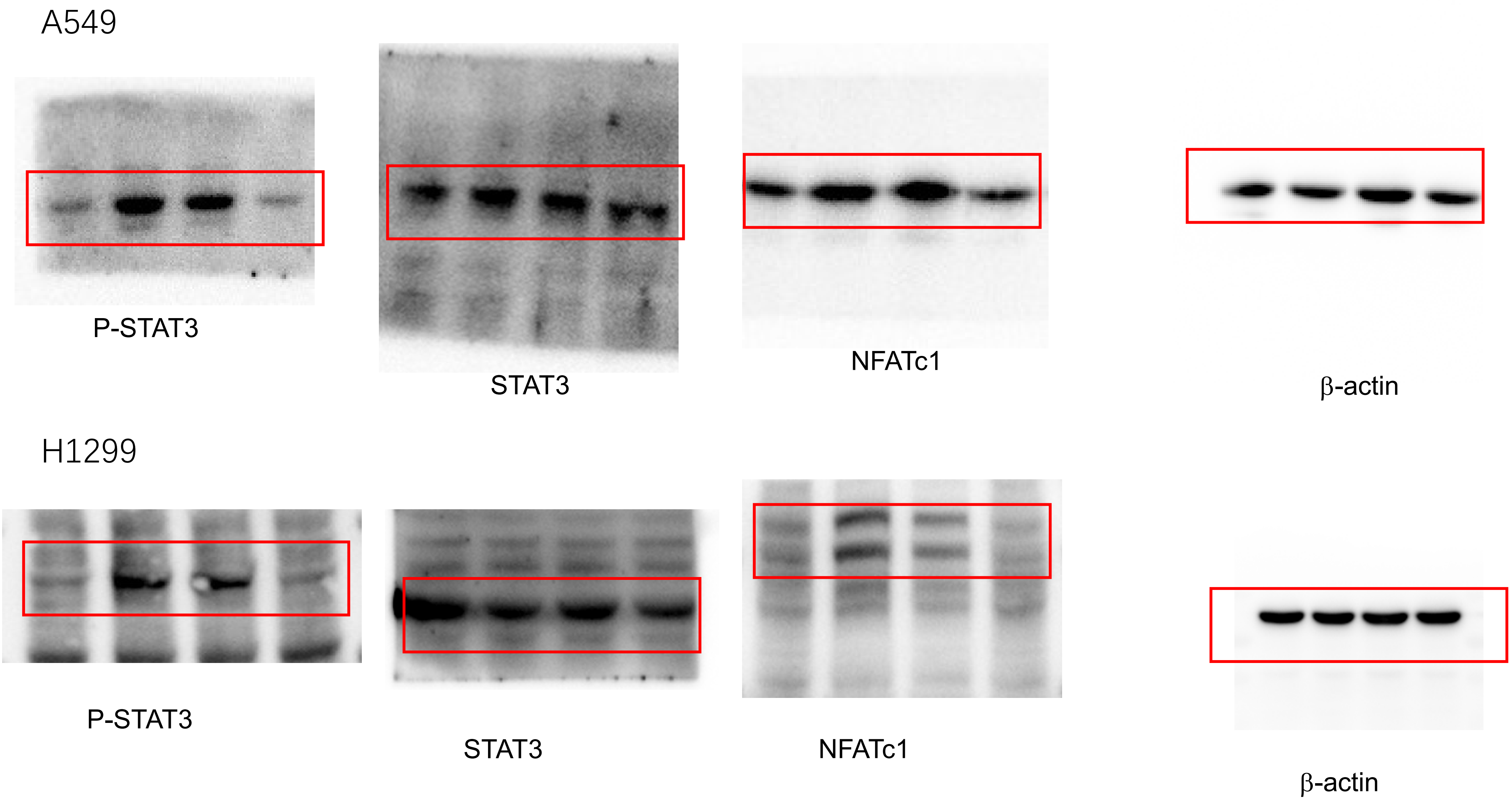
A549
P-STAT3
STAT3
NFATc1
β-actin
H1299
P-STAT3
STAT3
NFATc1
β-actin

Figure 6C

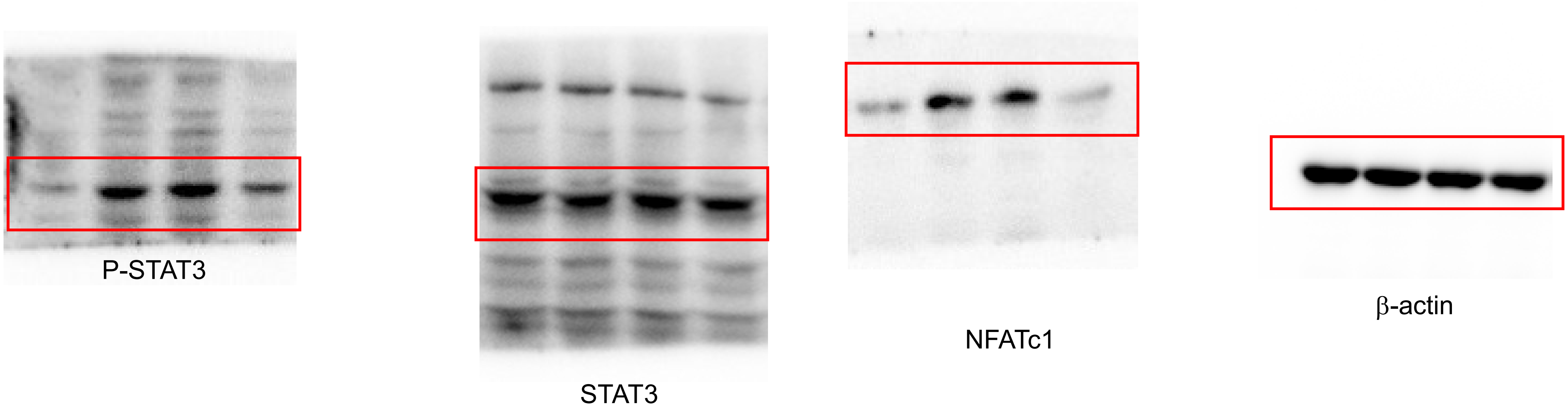

Supplement: Supplementary file 2 — Original Data File [file 41419_2023_5819_MOESM2_ESM.pdf]
